# Cation-Doped Amino-Functionalized Zirconium MOF Nanocrystals for Enhanced Photocatalytic Degradation of 1‑Naphthylamine

**DOI:** 10.1021/acs.langmuir.5c05475

**Published:** 2025-12-26

**Authors:** Ola Haidar, Hassan Wehbe, Thibault Roques-Carmes, Joumana Toufaily, Mohamad Hmadeh

**Affiliations:** † Department of Chemistry, 11238 American University of Beirut, Riad El-Solh, P.O. Box 11-0236, Beirut 1107 2020, Lebanon; ‡ Laboratoire Réactions et Génie des Procédés, UMR 7274 CNRS, 137665 Université de Lorraine, 54000 Nancy, France; § Laboratory of Applied Studies for Sustainable Development and Renewable Energy (LEADDER), Doctoral School for Science and Technology (EDST), Lebanese University, Campus Rafic Hariri, Hadath 6573/14, Lebanon; ∥ Laboratory of Materials, Catalysis, Environment and Analytical Methods (MCEMA), Faculty of Science, Lebanese University, Campus Rafic Hariri, Hadath 6573/14, Lebanon

## Abstract

The development of efficient and practical photocatalysts is a critical area of research, particularly for environmental applications, such as water purification. Metal–organic frameworks (MOFs) have emerged as promising materials for photocatalytic reactions due to their tunable structures and high surface areas. In this study, eight different MOF samples including UiO-66, UiO-66-NH_2,_ and their doped versions were synthesized by solvothermal and microwave-assisted methods and fully characterized using XRD, TGA, BET, SEM-EDX, and XPS. These materials were applied as photocatalysts for the degradation of 1-naphthylamine (1-NA) under visible light irradiation. The doped versions, (Ti–Zr)-UiO-66-(NH_2_) and Fe-UiO-66-(NH_2_), exhibited enhanced visible light absorption and reduced band gaps compared with the pristine UiO-66. Thus, 15%(Ti–Zr)-UiO-66-(NH_2_) and Fe-UiO-66-(NH_2_) achieved 75% and 65% degradation of 1-NA in 1 h, respectively.. The improved photocatalytic performance was attributed to (i) the functionalization with amino groups, which altered the electronic structure by narrowing the band gap, and (ii) doping with Ti^4+^ and Fe^3+^, which improved catalytic activity and reaction efficiency. Titanium doping introduced oxo-bridged hetero–Zr-Ti clusters, while iron doping facilitated a metal-to-cluster charge transfer (MCCT) process, enhancing the optical and catalytic properties of the MOFs. These findings highlight the potential of structural tuning of UiO-66-based MOFs for enhanced photocatalytic degradation of micropollutants in water under visible light, offering a sustainable and energy-efficient solution for water treatment.

## Graphical abstract

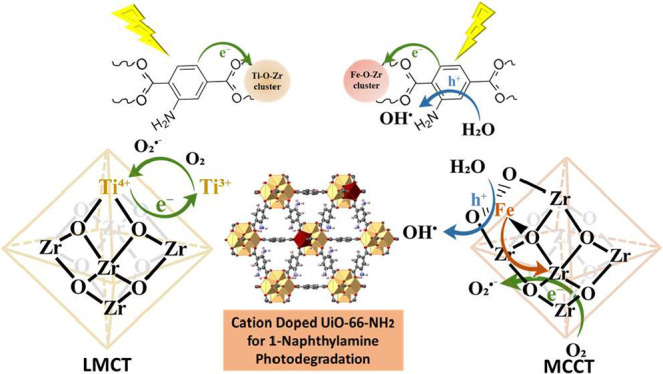

## Introduction

1-Naphthylamine (1-NA), also known as 1-amino-naphthalene, serves as a key chemical intermediate in various industrial applications.^1–3^ It is widely used as a coupling agent in the production of azo dyes, herbicides, and rubber antioxidants such as phenyl-alpha-naphthylamine. Additionally, 1-NA is employed in the manufacturing of rubber-covered cables, paints, pigments, plastics, and toning prints.^4–6^ Other potential sources of 1-NA include waste byproducts from direct coal liquefaction processes and destructive distillation methods commonly used in oil refining and petrochemical production. Among these, the dyestuff industry is considered the most significant contributor to environmental 1-NA pollution, as azo compounds account for approximately 60% of all organic dyes currently in use.^3,7,8^ The increasing demand for rubber products contributes to higher levels of contaminants in wastewater, highlighting the urgent need for effective removal methods to protect the environment and public health. Various methods have been investigated for the removal of 1-NA, including adsorption and advanced oxidation processes (AOPs), such as Fenton reactions and sulfate radical-based oxidations (SO_4_^·–^).^9,10^

Adsorption is a straightforward approach; however, its primary limitation lies in its nondestructive nature. AOPs generate highly reactive oxygen species that can break down organic pollutants into less harmful byproducts. Under optimized conditions, these processes can even mineralize contaminants to carbon dioxide and water. Among AOPs, photocatalysis is particularly ecofriendly, especially when solar energy is utilized. Traditional photocatalysts like TiO_2_, ZnO, and Fe_2_O_3_ have shown success in pollutant degradation; however, their broader application is often hindered by limited visible light absorption and the rapid recombination of photoinduced electron–hole pairs, which reduces efficiency^11,12^

More recently, metal–organic frameworks (MOFs), which are constructed by joining metal clusters and organic linkers, have gained growing attention as photocatalysts due to their tunable porous structures, large surface areas, and numerous active sites^13–15^ The structural properties of MOFs can be tailored by altering the metal centers or modifying the organic linkers with various functional groups, optimizing their performance for targeted applications.^16–19^ Similar to metal oxide semiconductors, MOFs can be photoexcited to generate electron–hole pairs.^20^ Additionally, the organic ligands in MOFs have strong light absorption capabilities, thanks to large π-conjugated systems.^21^ Due to these unique properties, MOFs are increasingly studied for the photocatalytic degradation of organic pollutants.^22^ Moreover, catalyst loss remains a significant challenge during photocatalysis reactions. Thus, adding robust MOFs as heterogeneous catalysts into the reaction system offers an effective solution to minimize the risk of secondary pollution. However, certain MOFs are susceptible to hydrolysis when exposed to water, as the weak coordination bonds between metal ions and oxygen can lead to irreversible structural collapse.^23^ This instability prevents the application of many MOF materials in processes involving aqueous media. Nevertheless, UiO-66-based MOFs, one of the MOFs which stands out for its high thermal stability and strong chemical resistance to water, are attributed to the robust interactions between Zr (IV) atoms and carboxylate oxygens.^24^ Despite these advantages, UiO-66 requires ultraviolet radiation to photoactivate it, due to its relatively wide band gap (∼3.60 eV).^25^ To enhance its practical use, recent reports focus on modifying the UiO-66 structure through different approaches to achieve a narrower band gap for visible light activation, enabling the use of solar energy.

One effective approach to enhance the visible light absorption of MOFs is through functionalizing organic ligands with specific groups to tailor the band gap.^26^ For example, modifying UiO-66 with sulfur-based functional groups such as sulfonate, thiol, and methylthio has been shown to lower the band gap to values of 2.80, 3.02, and 3.22 eV, respectively. Similarly, a series of UiO-66 variants with bromine, amino, thiol, and hydroxyl groups were synthesized, achieving band gaps ranging from 3.91 to 2.69 eV, which improved their performance in dye degradation under visible light.^27^ Moreover, doping the UiO-66 cluster with metals such as Ni, Co, Fe, Ce, and Ti can alter the energy band structure, introducing new energy levels that narrow the band gap. This modification broadens the absorption range in the visible spectrum and improves the photogenerated carrier production^28,29^ Additionally, incorporating metals into the MOF framework enables the formation of bimetallic-centered MOFs, where the doped metal acts as an electron mediator, facilitating charge transfer and boosting photocatalytic efficiency.^30^

In this work, Ti- and Fe-doped amino-functionalized UiO-66 were synthesized using solvothermal and microwave-assisted methods, respectively, resulted in the formation of (Ti–Zr)-UiO-66-(NH_2_) and Fe-UiO-66-(NH_2_) nanocrystals as shown in Scheme 1. Because Ti^4^^+^ is chemically similar to Zr^4^^+^, it can be incorporated into the UiO-66 framework without disrupting its structure. Introducing the smaller Ti^4^^+^ cation slightly modifies the local environment within the pores and influences framework properties, and controlled Ti incorporation has been shown to alter the structure of the material and electronic characteristics in a beneficial way.^31^ Similarly, Fe-based modifications have also attracted considerable interest, as iron offers favorable characteristics such as low cost, low toxicity, multiple accessible oxidation states, and overall environmental compatibility. These intrinsic properties make Fe a suitable element for introducing additional redox versatility and enhancing the behavior of UiO-66 when incorporated under appropriate conditions.^32,33^ The photocatalytic activities of these MOFs were assessed for the degradation of 1-NA under visible light irradiation. Furthermore, the effects of functional group modifications and metal ion doping on the structural and catalytic characteristics of the MOFs were systematically explored. To the best of our knowledge, no prior studies have investigated the photodegradation of 1-NA using functionalized and doped MOFs.

**Scheme 1:**
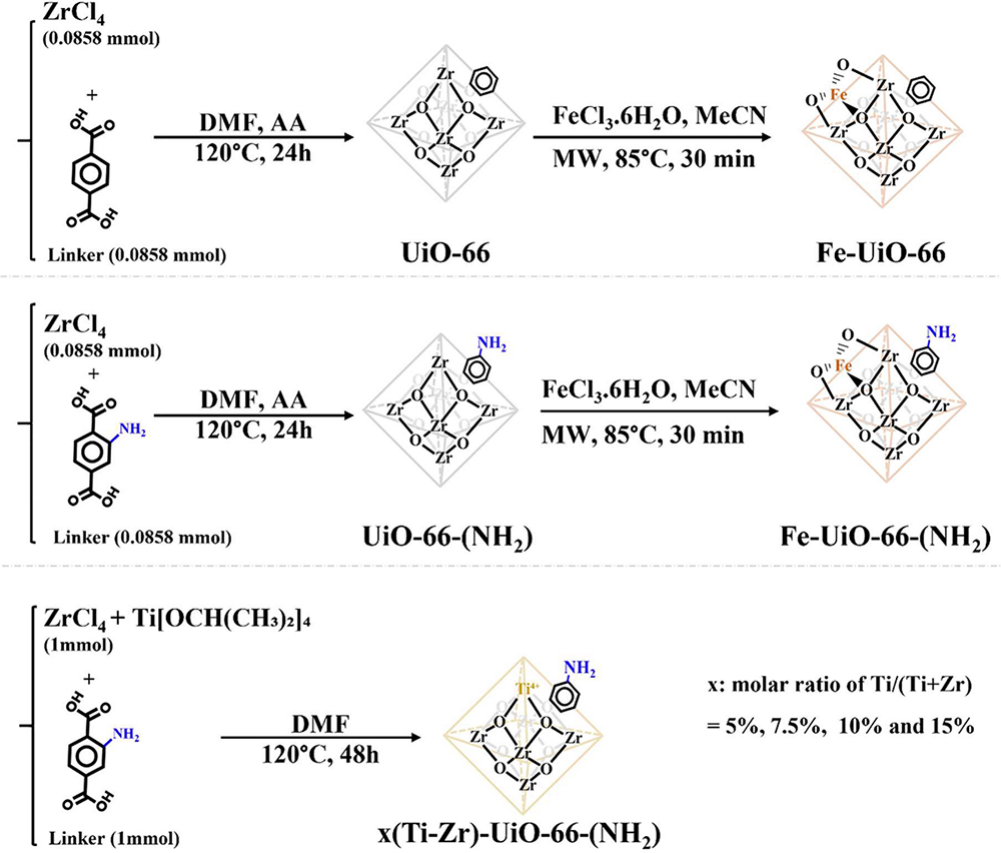
Schematic Illustration Outlining the Synthesis Procedure Utilized in the Preparation of the MOF Catalysts Investigated in This Study

## Materials and Methods

All chemicals used in this study were obtained from commercial suppliers and used without additional purification (see S1 for details). X-ray diffraction patterns were obtained on a Bruker D8 advance X-ray diffractometer with Cu Kα radiation (λ = 1.5418 Å). Gas sorption measurements were performed by using a Micromeritics Gemini VII 2390p adsorption analyzer. Scanning electron microscopy (SEM) was carried out with a scanning electron microanalyzer (Tescan MIRA3 scanning electron microscope). The energy-dispersive X-ray spectroscopy (EDX) was also performed to confirm the presence of doped elements on the photocatalysts. The UV–visible absorbance and the UV–visible diffuse reflectance spectra (DRS) of the powdered samples were obtained by using a Shimadzu 2600–2700 spectrometer. The photoluminescence (PL) was performed by using a fluorescence spectrometer. X-ray photoelectron spectroscopy (XPS) was used to identify the elemental composition of the doped samples and the oxidation states of the elements using a KRATOS AXIS Ultra DLD XPS/UPS spectrometer.

### Preparation of UiO-66 and UiO-66-(NH_2_)

In a typical synthesis, 0.0858 mmol of ZrCl_4_ and 0.0858 mmol of the organic linker were dissolved in 10 mL of DMF containing 1.83 mL of acetic acid. This solution was sealed in a 20 mL glass vial and heated at 120 °C for 24 h. The resulting solid was separated by centrifugation, followed by washing with DMF and methanol. To remove residual DMF, the product was immersed in methanol for 2 days to exchange out the DMF and then dried under vacuum at 60 °C overnight.^34^ The photograph of the macroscopic aspect of the final MOF is reported in Figure S1.

### Preparation of (Ti–Zr)-UiO-66-(NH_2_)

Ti-doped UiO-66-(NH_2_) was synthesized by dissolving varying amounts of titanium isopropoxide (Ti­[OCH­(CH_3_)_2_]_4_, used as a Ti^4+^ precursor), ZrCl_4_, and 1 mmol of 2-aminoterephthalic acid (H_2_BDC-NH_2_) in 50 mL of DMF within a Teflon-lined stainless-steel autoclave. The exact Ti amounts were adjusted to give Ti/(Ti+Zr) molar ratios of 5, 7.5, 10, and 15%, corresponding to 0.05, 0.075, 0.10, and 0.15 mmol of Ti precursor, respectively (with the total metal content fixed at 1 mmol). The autoclave was sealed and heated at 120 °C for 48 h. After cooling to room temperature, the solid product was separated by centrifugation and washed three time with DMF and centrifuged and then washed three times with anhydrous methanol. The obtained yellowish particles were dried under vacuum at 120 °C overnight. The samples were labeled as 5%(Ti–Zr)-UiO-66-(NH_2_), 7.5%(Ti–Zr)-UiO-66-(NH_2_), 10%(Ti–Zr)-UiO-66-(NH_2_), and 15%(Ti–Zr)-UiO-66-(NH_2_).^35^ The photograph of the macroscopic aspect of the final MOF product is reported in Figure S1.

### Preparation of Fe-UiO-66 and Fe-UiO-66-(NH_2_)

Typically, 40 mg of the as-prepared UiO-66 or UiO-66-(NH_2_) and 80 mg of iron­(III) chloride hexahydrate (FeCl_3_·6H_2_O) were dispersed in 4 mL of MeCN by using ultrasonic agitation. This mixture was then transferred to a 7 mL microwave vessel, sealed, and heated to 85 °C in a microwave reactor at a power of 315 W and a pressure of 9 bar, maintaining this temperature for 30 min with constant stirring (600 rpm). After being cooled to room temperature, the brownish solid was separated by centrifugation and washed with H_2_O and methanol to remove excess Fe^3^^+^ ions. The Fe-UiO-66 and Fe-UiO-66-(NH_2_) products were then dried at 60 °C under vacuum.^34^ Different Fe:Zr ratios were tested; however, the amount of Fe incorporated remained unchanged, suggesting that Fe is likely fixed at the defect sites within the UiO-66 structure. The photograph of the macroscopic aspect of the final MOF is reported in Figure S1.

### Evaluation of Photocatalytic Activity

The degradation tests for 1-NA in aqueous solution were conducted at 25 °C in a 500 mL Pyrex glass jacketed reactor, using a 100 W halogen lamp for visible light irradiation at pH = 6.5 (Figure S4). A photocatalyst suspension (25 mg) was prepared in an aqueous 1-NA solution (250 mL, 5 mg/L) and stirred in the dark for 1 h to establish the adsorption equilibrium.^9^ Samples of 0.45 mL were taken at regular intervals during the reaction and filtered with 0.2 μm PTFE filters (Whatman) to remove the photocatalyst. The concentration of 1-NA was analyzed by HPLC (System G1380AA) with a C18 column (250 × 4.6 mm, 5 μm) by using a gradient elution method. The mobile phase consisted of a mixture of acetonitrile, water, and H_2_SO_4_ in a volume ratio of 0.35:0.65:0.01, with a flow rate of 1.0 mL/min and an injection volume of 10 μL. The absorbance detector was set to 308 nm and the maximum absorbance wavelength of 1-NA as determined by spectrophotometric analysis over a range of 200–900 nm. Under these conditions, the retention time of 1-NA was approximately 3.25 min. Each experiment was repeated twice with a standard deviation of less than 5%.

The photocatalytic activity was reported as *C*/*C*_0_ versus time of irradiation. However, the photodegradation capacity percentage (% degradation) can be calculated using the following equation:

$$\%\mathrm{Degradation}=\left(\frac{C_{0}-C}{C_{0}}\right)\times 100$$ 1

where:

- *C*_0_ (mg/L) is the initial concentration of 1-NA before exposure to light.
- *C* (mg/L) is the concentration of 1-NA at a specific time *t* during or after the photodegradation process.

The effects of 1-NA concentration, catalyst dosage, and pH were investigated, with all experiments conducted at room temperature. The 1-NA concentration was varied between 5 and 15 mg/L using 25 mg of catalyst, while the catalyst dosage ranged from 15 to 45 mg in 250 mL of an initial 1-NA solution at 5 mg/L. For the pH experiments, the pH was adjusted to values between 2 and 11 using 1 M HCl and 2 M NaOH solutions to precisely control the acidity or basicity of the medium. Twenty-five mg of catalyst was dispersed in 250 mL of an initial 1-NA solution at a concentration of 5 mg/L. To ensure adsorption equilibrium, the suspensions were stirred in the dark for 1 h. During the reaction, 0.45 mL samples were collected at regular intervals for 60 min and filtered through 0.2 μm PTFE filters to separate the photocatalyst. The 1-NA concentration was then measured by using UV–visible spectroscopy.

Recycling experiments were conducted to evaluate the stability and reusability of the 15%(Ti–Zr)-UiO-66-(NH_2_) and Fe-UiO-66-(NH_2_) materials, which were found to be the best performing samples. After each photodegradation cycle, the samples were collected and reused. The used MOF was immersed in 10 mL of ethanol and stirred continuously for 2 h. The recovered materials were then dried at 110 °C for 12 h. These regenerated MOF samples were subsequently tested in 250 mL of a 1-NA contaminated solution with an initial concentration of 5 ppm. This procedure was repeated five times on the same sample to assess its reusability.

### Free Radical Capture Experiments

In the free radical scavenging experiments, benzoic acid, 2-propanol, AgNO_3_, and triethylamine (TEA) were used as scavengers for superoxide radicals (O_2_^·–^), hydroxyl radicals (OH^·^), electrons (e^–^), and holes (h^+^), respectively.^36^ These experiments followed the same protocol as the photocatalytic degradation tests with the addition of 1 mM of each scavenger to the reaction system. Aliquots of the reaction mixture were collected at 5 min intervals, filtered using 0.2 μm PTFE filters to remove the photocatalyst, and then analyzed via UV–vis spectroscopy.

## Results and Discussion

### Structure and Morphology

The powder X-ray diffraction (PXRD) patterns of the synthesized samples are shown in Figure 1. UiO-66 and UiO-66-(NH_2_) display well-defined and intense diffraction peaks, closely matching the simulated pattern of UiO-66 which reflects their high crystallinity and phase purity. For Ti-doped UiO-66 MOFs, XRD patterns exhibit only the characteristic diffraction peaks of pristine UiO-66, with no additional peaks corresponding to Ti^4^^+^ oxides or Ti-related impurities. The diffraction peak at approximately 2θ = 7.5° shifts to lower angles as the Ti/Zr ratio increases. This shift suggests that the titanium species are not simply grafted onto or encapsulated within the UiO-66 framework. Instead, they likely integrate with Zr to form oxo-bridged hetero-Zr–Ti clusters. This result provides preliminary evidence that the MOFs were successfully doped with Ti ions.^35^ Additionally, the broader peaks in the doped samples suggest a decrease in the particle size, as later confirmed by SEM images. For Fe-postmetalated UiO-66 MOFs, the XRD patterns display sharp and intense peaks, indicating that their highly crystalline nature was preserved. The incorporation of Fe does not induce an appreciable shift of the characteristic UiO-66 diffraction peaks. Additionally, the patterns closely resemble the simulated XRD pattern of UiO-66, suggesting that the incorporation of Fe did not alter the crystal structure and that Fe was well-dispersed within the framework. Since Fe cations were postsynthetically added to the MOF structure, it was unlikely they substitute Zr cations within the cluster; however, they were incorporated in the defected sites.^37^

**Figure 1:**
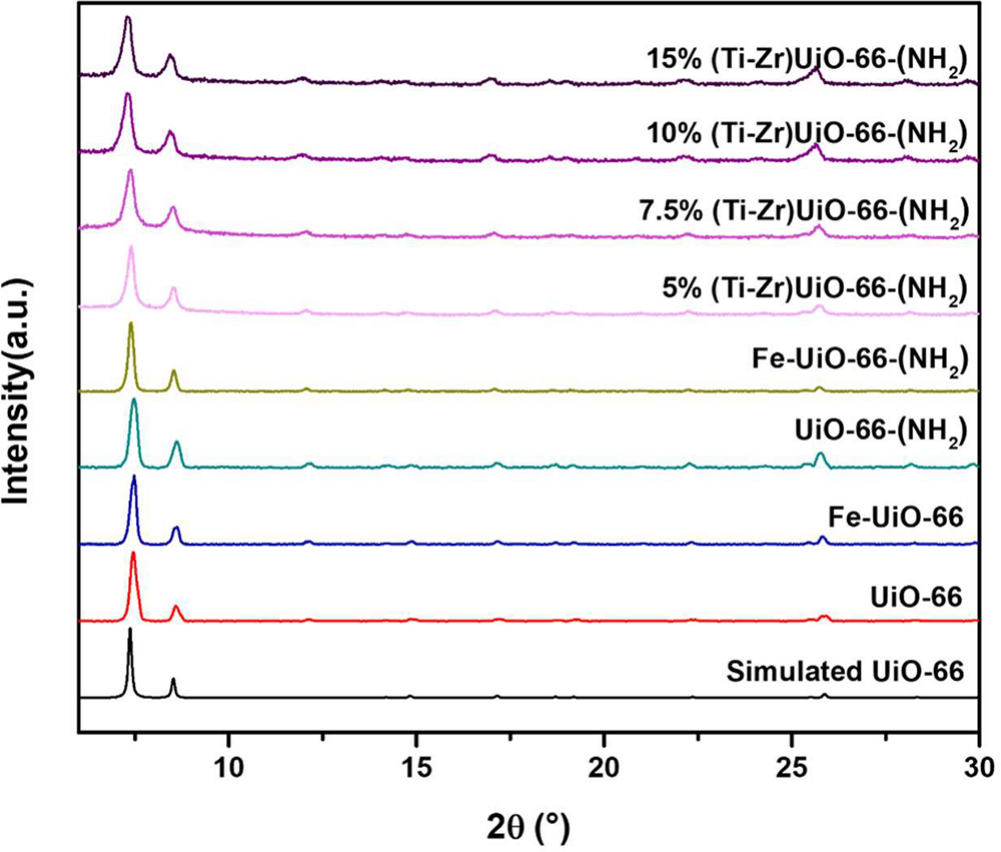
PXRD patterns of the UiO-66-MOF samples.

The FT-IR spectra of all samples are depicted in Figure S2. All the synthesized MOFs show intense bands at 1583 cm^–1^ and at approximately 1400 cm^–1^, corresponding to the asymmetric and symmetric stretching vibrations of the carboxylate groups from the terephthalic linkers. A weaker band at 1507 cm^–1^, associated with the C=C vibrations of the benzene ring, which is part of the BDC linker, confirms the presence of dicarboxylic acid linkages in the doped UiO-66 MOFs. In the low-frequency region, the band near 577 cm^–1^ is attributed to the asymmetric stretching vibrations of Zr–(OC), while the peaks around 666 and 475 cm^–1^ correspond to the stretching vibrations of Zr–Oμ_3_–O and Zr–Oμ_3_–OH bonds of the Zr_6_ cluster, respectively.^38^ The intensity of these bands decreases in the doped UiO-66 MOFs, particularly with a higher titanium content, suggesting the substitution of Zr by Ti within the cluster. For UiO-66-(NH_2_), the band between 3200 and 3500 cm^–1^ is broader than that of UiO-66, likely due to the stretching vibrations of the −NH_2_ group at 3300 cm^–1^ overlapping with the characteristic bands of adsorbed water. Additionally, UiO-66-(NH_2_) displays a characteristic C–N stretching vibration band at 1260 cm^–1^, which is attributed to the C–N bonds of the amino groups attached to the benzene ring.^39^ In the case of Ti-doped UiO-66, no distinct peaks corresponding to Ti–O bonds are observed. This could be due to the overlap of Ti–O vibrations with Zr–O bands in the low-frequency region (∼450–700 cm^–1^), making them indistinguishable.^40,41^ Furthermore, the titanium content in the samples may be too low to produce detectable Ti–O bands in the FTIR spectrum. For Fe-doped UiO-66, the peak at 520 cm^–1^ corresponds to the Fe–O bond vibrations, confirming the incorporation of iron into the MOF structure.^42^

X-ray photoelectron spectroscopy (XPS) was employed to investigate the elemental composition of UiO-66, UiO-66-(NH_2_), 15% (Ti–Zr)-UiO-66-(NH_2_), and Fe-UiO-66-(NH_2_). The survey spectra (Figure 2a) confirmed that carbon, oxygen, and zirconium were the principal elements present in all samples (Table S1). However, the incorporation of Ti and Fe introduced noticeable changes in the crystal lattice, as evidenced by the shifts in the obtained binding energies. In addition to identifying the surface composition, XPS analysis provided insights into the oxidation states of the doped metals in 15% (Ti–Zr)-UiO-66-(NH_2_) and Fe-UiO-66-(NH_2_). Comparison with the spectrum of UiO-66 and UiO-66-(NH_2_) revealed that the introduction of Ti cations leads to characteristic Ti 2p signals (Figure 2c). These features correspond to the spin–orbit doublets of Ti 2p_3/2_ at approximately 458.9 eV.^35^ The presence of these peaks is characteristic of the Ti^4^^+^ oxidation state, confirming the successful incorporation of the Ti element within the MOF cluster. Furthermore, upon the introduction of Ti and Fe species, the Zr 3d_5/2_ peak shifts to a higher binding energy from 182.81 to 182.99 eV for 15% (Ti–Zr)-UiO-66-(NH_2_) and to 182.97 eV for Fe-UiO-66-(NH_2_) (Figure 2b). The incorporation of Fe into UiO-66-(NH_2_) gives rise to characteristic Fe 2p signals (Figure 2d). The spectrum displays two components, Fe 2p_3/2_ and Fe 2p_1/2_, arising from spin–orbit splitting, positioned at 711.25 and 724.04 eV, respectively.^43^ These features indicate the coexistence of the Fe^3^^+^ and Fe^2^^+^ species. Because Ti (IV) and Fe (III) have a stronger electron absorption capacity than protons, Zr­(IV)–O-Ti­(IV) and Zr­(IV)–O-Fe­(III) were formed by this transformation. Thus, the Ti^4+^ and Fe^3+^ ions in 15% (Ti–Zr)-UiO-66-(NH_2_) and Fe-UiO-66-(NH_2_) were connected to the Zr oxygen cluster through Ti/Fe–O–Zr linkages. The C 1s, O 1s, and N 1s XPS spectra have been included in the Supporting Information (Figure S3). The C 1s spectrum (Figure S3a) shows three peaks at 284.8, 286.01, and 288.82 eV, corresponding to C–C, C–O, and the O–C=O groups, respectively. After functionalization with amine groups, the formation of C–N bonds shifts the C–O peak to 286.08 eV, indicating the presence of −NH_2_. In the O 1s spectrum (Figure S3b), three signals are observed and attributed to Zr–O–Zr (530.25 eV), Zr–OH (533.41 eV), and Zr–O–C (531.86 eV), reflecting the oxygen environments within the Zr_6_-oxo cluster, i.e., Zr_6_-oxo nodes, exposed hydroxy groups, and BDC linkers present within the UiO-66, respectively.^44^ The formation of Ti–O bonds causes a shift of the Zr–O–Zr peak to lower binding energies, which may be attributed to an increase in the electron density around the oxygen atoms. This suggests that titanium cations are incorporated into the UiO-66-(NH_2_) framework as electron donors through the formation of oxo-bridged heterometallic Zr–Ti clusters.^45^ Concerning Fe-doped sample, it can be observed that the shift of the O 1s peak after Fe doping suggested the formation of Fe–O bonds as Fe^3+^ cations could potentially bond to the Zr_6_ clusters of MOF.^37^ The N 1s spectrum (Figure S3c) shows a peak at 399.46 eV, confirming the presence of amino groups in the material.

**Figure 2:**
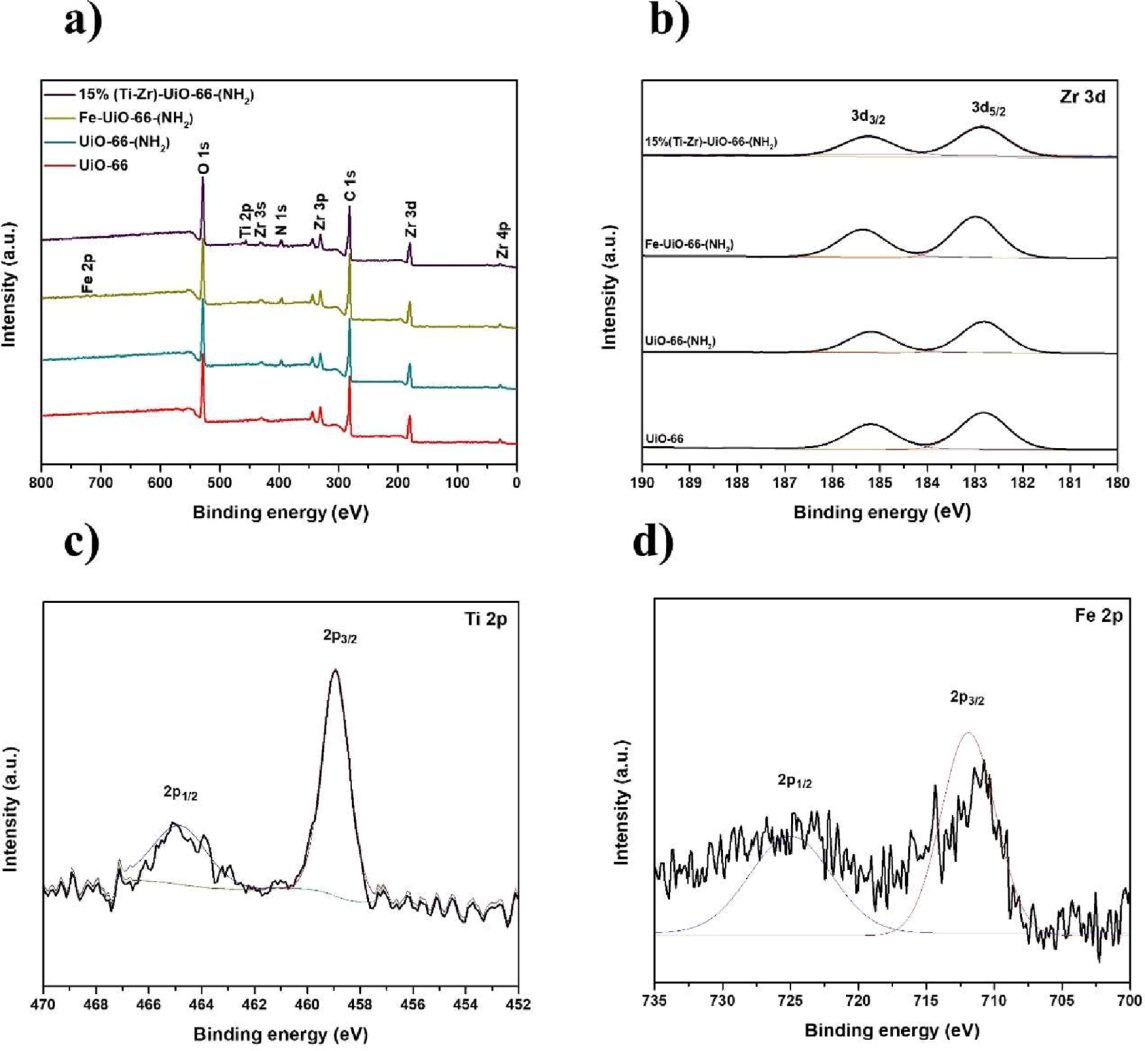
XPS spectra of UiO-66, UiO-66-(NH_2_), 15% (Ti–Zr)-UiO-66-(NH_2_), and Fe-UiO-66-(NH_2_): (a) survey scan, (b) Zr 3d, (c) Ti 2p XPS in 15%(Ti–Zr)-UiO-66-(NH_2_), and (d) Fe 2p in Fe-UiO-66-(NH_2_).

The morphological characteristics of nondoped and Ti- and Fe-doped UiO-66 MOFs were examined using SEM, as shown in Figure 3. Both UiO-66 and UiO-66-(NH_2_) demonstrated octahedral crystallite morphologies. The Ti-doped UiO-66 MOFs retained a morphology similar to that of UiO-66, characterized by octahedral-shaped crystals with a smaller particle size (Table S2), likely due to the effects of Ti doping. With an increasing Ti content, a reduction in crystallinity is observed, accompanied by increased particle agglomeration, which is in agreement with what was observed in the PXRD patterns. For Fe-doped UiO-66, the introduction of Fe^3^^+^ by microwave-assisted postmetalation did not alter the original octahedral morphology of the UiO-66 framework. Additionally, no extra particles were observed on the Fe-doped-UiO-66 surface, confirming the successful incorporation of iron into the MOF cluster. This observation is consistent with the FT-IR spectroscopy results, indicating that Fe-doped-UiO-66 was effectively synthesized using a microwave-assisted method. Moreover, the EDX spectra confirm the successful incorporation of Ti and Fe elements into the doped UiO-66 samples (Table S1).

**Figure 3:**
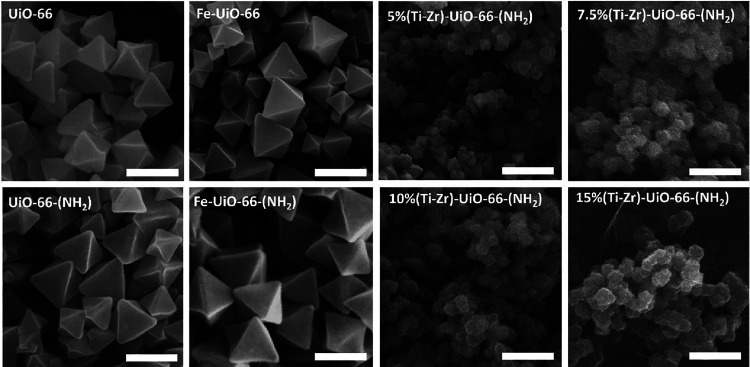
SEM images of UiO-66, UiO-66-(NH_2_), Fe-doped UiO-66 (scale 200 nm), and Ti-doped UiO-66 MOFs samples (scale bars 500 nm).

The surface area and porosity of the samples were analyzed using N_2_ adsorption–desoprtion isotherms, and the obtained results are illustrated in Figure 4a. The BET surface areas and pore volumes are reported in Table S2. UiO-66 and UiO-66-(NH_2_) show type I adsorption isotherms, characteristic of microporous materials, with specific surface areas of 1066 and 972 m^2^/g, respectively. The incorporation of functional groups into the backbone of the MOFs leads to a decrease in the specific surface area compared to that of pristine UiO-66. For Ti-doped-UiO-66 MOFs, all samples exhibited type I isotherms, as well. The BET surface area showed a consistent decrease with increasing amount of Ti doping from 976 m^2^/g at 5% to 916 m^2^/g at 15% Ti. This reduction in *S*_BET_ is likely due to the loss of the crystallinity and porous structure of the framework upon Ti incorporation, as confirmed by PXRD analysis. For Fe-doped MOFs, the nitrogen sorption isotherms revealed that the adsorption capacity was slightly lower than that of undoped UiO-66 MOFs. The BET surface area reached 920 m^2^/g for Fe-UiO-66 and 823 m^2^/g for Fe-UiO-66-(NH_2_). This reduction may result from mass occupation by the incorporated Fe^3^^+^ ions within Zr clusters.

**Figure 4:**
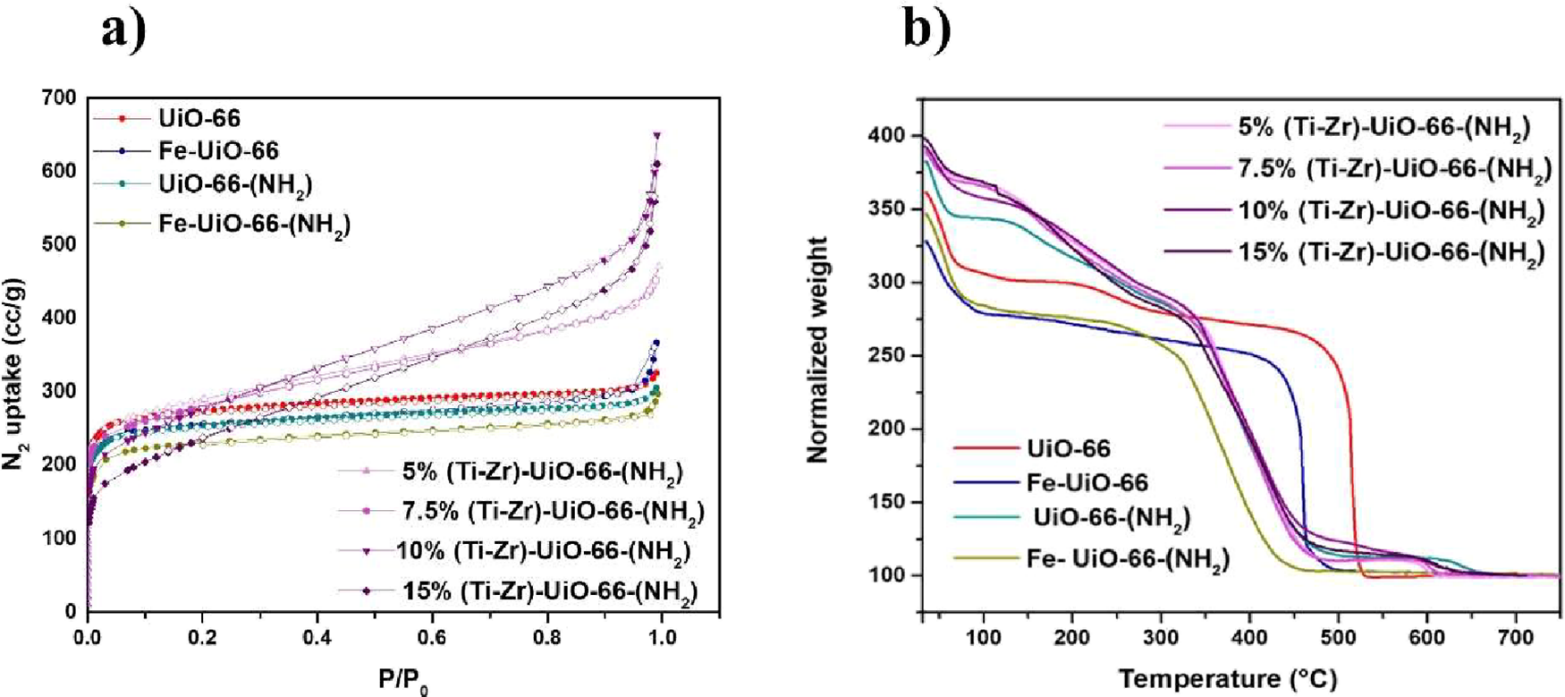
(a) N_2_ adsorption–desorption isotherms and (b) TGA curves of the UiO-66 MOFs samples.

The pore volume values follow a trend consistent with those of BET surface areas (Table S2). For pristine UiO-66, the pore volume reaches 0.50 cm^3^/g, which decreases upon Fe incorporation (0.40 cm^3^/g), reflecting the partial occupation of the porous network by Fe^3^^+^ ions within the Zr clusters. Interestingly, UiO-66-(NH_2_) exhibits a slightly higher pore volume (0.54 cm^3^/g) in comparison to UiO-66, which can be attributed to the steric effects of the amino functional groups modifying the packing environment of the linkers. However, Fe doping in UiO-66-(NH_2_) reduces the pore volume to 0.50 cm^3^/g, again indicating that Fe^3^^+^ ions partially block the pore channels. In contrast, Ti-doped UiO-66-(NH_2_) samples exhibit pore volumes significantly higher than those of their undoped counterparts, reaching a maximum of 0.72 cm^3^/g at 5% Ti content. This increase suggests that Ti substitution for Zr creates additional structural defects or irregularities, which enlarge the available pore volume. With further Ti incorporation, the pore volume gradually decreases (0.70 at 7.5% Ti, 0.65 at 10% Ti, and 0.55 cm^3^/g at 15% Ti), consistent with the progressive distortion and partial collapse of the porous framework, as also evidenced by the decrease in the surface area.

Thermogravimetric analysis (TGA) has been widely recognized as a valuable technique for gaining first-hand information into the presence of structural defects.^46^ The TGA curves were presented in Figure 4b and reveal three distinct phases of weight loss. The first phase, occurring between 35 and 100 °C, corresponds to the removal of adsorbed water, a trend observed in all of the MOFs. The second phase, extending from 100 °C to the specific temperature plateau (Temp p) for each MOF, involves the dehydroxylation of the zirconium cluster and the removal of monocarboxylate linkers. For the doped samples, this plateau is lower than that of ideal UiO-66, suggesting the presence of defects sites where fewer than 12 BDC linkers are coordinated to each cluster, resulting in a lower molecular weight than Zr_6_O_6_(BDC)_6_. The third stage corresponds to the framework destruction phase. The number of missing linker defects was calculated from TGA and presented in Table S2. The UiO-66 initially modulated with acetic acid exhibits a missing linker number of 0.87. The introduction of amino groups increases the number of defects, raising it to 1.22, indicating that the modulated synthesis promotes defect formation. Postsynthetic metalation with Fe does not change this number much. However, in situ doping with Ti leads to highly defective structures, with the 15% (Ti–Zr)-UiO-66-(NH_2_) showing a missing linker number of 2.03.

### Optical Properties

The UV–vis absorption spectra of all MOF samples show a characteristic peak around 250 nm (Figure 5a), attributed to the Zr-oxo clusters in the framework, while a band centered around 400–420 nm is observed in UiO-66-(NH_2_), extends the absorption into the visible range due to NH_2_ functionalization. The latter introduces new electronic states that enhance visible light absorption, influencing the photocatalytic properties of the material. Interestingly, the UV–vis light absorbance of UiO-66-(NH_2_) increases upon Ti- and Fe-doping, particularly with higher amounts of Ti species. Enhanced UV–vis light absorption by the photocatalysts is anticipated to generate more photogenerated electrons and holes, which positively impact their photocatalytic activity.

**Figure 5:**
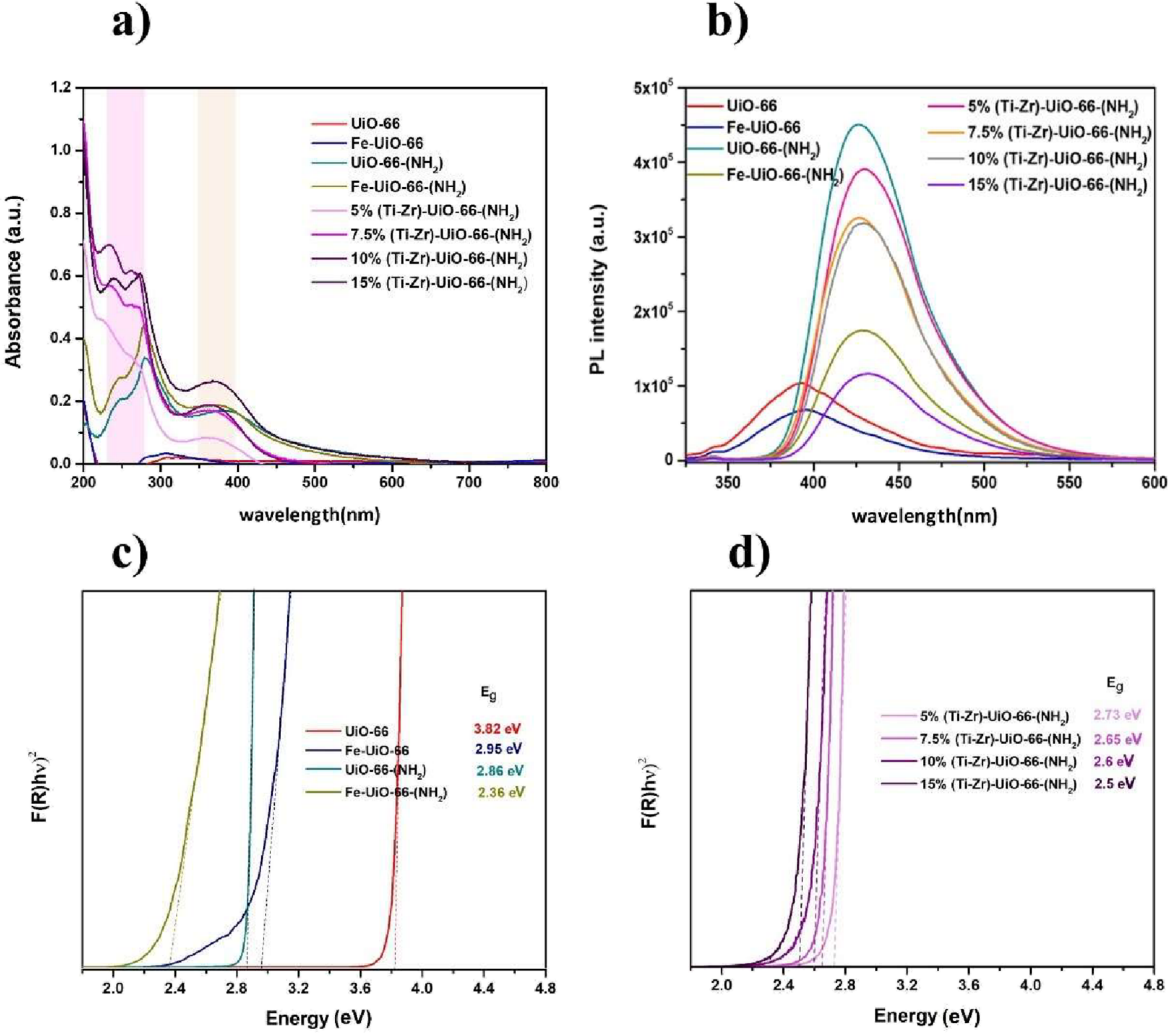
(a) UV–vis absorption spectra, (b) PL spectra, and (c,d) optical band gap determined by the Tauc equation of UiO-66 MOFs samples.

PL measurements are conducted to evaluate the recombination of photogenerated electron–hole pairs and to understand the photocatalytic behavior of the materials. The PL spectra are shown in Figure 5b. For Ti-doped-UiO-66 samples, the PL intensity decreases with higher titanium content. Among the samples, 15%(Ti–Zr)-UiO-66-(NH_2_) exhibits the lowest PL intensity, reflecting a lowest recombination rate of electron–hole pairs.^45^ Similarly, the introduction of iron species resulted in a decrease in PL intensity, suggesting a lower recombination rate of electron–hole pairs.

Considering these MOFs as indirect semiconductors,^47^ the optical band gap energy (*E*_g_) values are calculated using the Tauc equation (α*h*υ = *A*(*h*υ – *E*_g_)^2^), (Figure 5c,d) based on UV–vis DRS data. By extrapolating the linear region of the (αhυ)^1/2^ versus hυ curves to the *x*-axis, we can determine the *E*_g_ values. Results show that introducing amino groups into the UiO-66 structure reduces the band gap from 3.7 to 2.8 eV (Table S3). The functional groups are attached to the aromatic ring function as antennas, improving the light absorption efficiency. Additionally, the incorporation of Ti^4^^+^ and Fe^3^^+^ ions further reduces the band gap. Notably, increasing the titanium content in 15%(Ti–Zr)-UiO-66-(NH_2_) sample leads to a band gap of 2.48 eV. This suggests that titanium and iron species act as electron mediator in UiO-66-(NH_2_).^48^ Results demonstrate that the introduction of functional groups and the metal doping reduces the band gap, therefore enhancing the visible light absorption, which is considered as a crucial parameter in photocatalysis.

### Photocatalytic Activity

Figure 6 illustrates the time-course evolution of 1-NA during photocatalytic reactions under visible light irradiation using the MOF samples. Prior to the reaction tests, the adsorption of 1-NA is evaluated, revealing the lowest values in all cases. The 1-NA control (without catalyst) showed negligible photolysis under visible light, with only ∼5% degradation after 60 min, confirming that 1-NA is not significantly degraded by light alone. UiO-66 shows no observable photocatalytic activity, similar to the control experiment performed under identical conditions but without a catalyst. In contrast, functionalization with amino groups significantly improved the degradation efficiency, with UiO-66-(NH_2_) achieving 40% degradation after 1 h. Furthermore, doping with Ti and Fe resulted in an increased photocatalytic activity. Among the doped samples, 15%(Ti–Zr)-UiO-66-(NH_2_) and Fe-UiO-66-(NH_2_) demonstrated superior performance, achieving 75 and 65% conversion of 1-NA after 1 h of irradiation, respectively. The experimental concentration vs time curves were fitted to a pseudo-second-order eq (Figure S5b). The kinetic constant values of 0.068 L mg^–1^ min^–1^ for 15%(Ti–Zr)-UiO-66-(NH_2_) and 0.034 L mg^–1^ min^–1^ for Fe-UiO-66-(NH_2_) indicate that 15%(Ti–Zr)-UiO-66-(NH_2_) exhibits a higher degradation rate compared to that of Fe-UiO-66-(NH_2_). This is in agreement with previous reports which showed that the functionalization of UiO-66 with amino and halogen groups significantly enhances its catalytic and photocatalytic properties.^49^ Amine groups improve phosphate-ester hydrolysis through Bro̷nsted basicity, while halogenated derivatives, especially UiO-66-I, show increased activity in degrading chemical warfare agents like DMNP (dimethyl 4-nitrophenyl phosphate) due to stronger polarizing effects.^50^ Further studies revealed that electron-donating ligands (e.g., NH_2_) improve photocatalytic oxidation performance by enhancing charge separation. Notably, UiO-66-NH_2_ demonstrated superior visible light photocatalytic degradation of acetaminophen, due to its reduced bandgap and increased generation of reactive species.^51^

**Figure 6:**
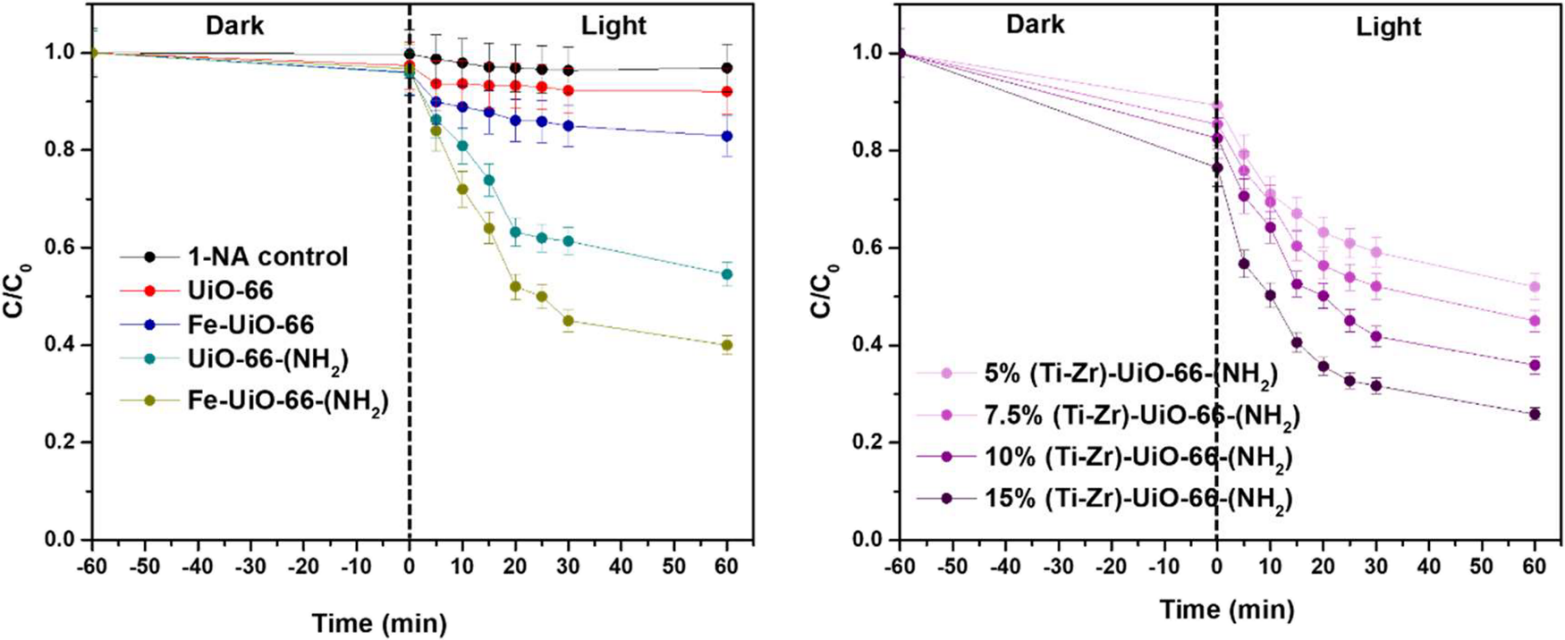
Visible photocatalytic degradation of 1-NA using the varying UiO-66 samples ([1-NA]_o_ = 5 mg L^–1^; *m*_catalyst_ = 25 mg; and light intensity = 100 W).

The construction of bimetal UiO-66 frameworks has been widely employed to enhance photocatalytic activity. Specifically, the doping of transition metal atoms with redox ability (e.g., Fe, Ti or Co) into the framework can significantly improve the photocatalytic efficiency for pollutant degradation.^52–55^ For instance, Co-doped UiO-66 synthesized via a one-step solvothermal method showed enhanced adsorption and photocatalytic efficiency for tetracycline removal.^56^ Similarly, Fe incorporation significantly boosted the peroxydisulfate (PS) activation ability of UiO-66, enabling efficient degradation of sulfonamide antibiotics.^54^ Moreover, Ti substitution in UiO-66-(NH_2_) led to a marked enhancement in both Cr­(VI) adsorption and its photocatalytic reduction to Cr­(III) under visible light irradiation.^55^

The introduction of NH_2_ groups into the terephthalic linker of UiO-66 MOFs remarkably affects their light absorption and band gap, as seen before, enhancing their photocatalytic performance under visible light. The electron-donating nature of the NH_2_ group facilitates additional electronic transitions, resulting in a red shift of the absorption edge and enabling visible light absorption, unlike the primarily UV absorption observed in pristine UiO-66. Furthermore, the NH_2_ functionalization decreases the band gap of UiO-66 from 3.7 to 2.8 eV, introducing new electronic states between the valence and conduction bands. This narrowed band gap allows for electron excitation under lower-energy visible photons, expanding the material’s photocatalytic activity spectrum. Similarly, doping UiO-66 MOFs with a second metal, such as Ti or Fe, further enhances their optical and electronic properties for visible-light-driven photocatalysis. Notably, the metal incorporated into the Zr-oxo cluster functions as an electron donor, contributing to the enhanced photocatalytic activity. The addition of Ti or Fe introduces metal-based electronic states and metal-to-cluster charge transfer (MCCT) transitions, respectively, improving light absorption in the visible region. Ti doping forms heterometallic oxo-clusters (e.g., Zr–Ti clusters) that reduce the band gap through synergistic electronic interactions. The better photocatalytic activity of 15%(Ti–Zr)-UiO-66-(NH_2_) can be due also to the presence of a greater number of structural defects that can provide extra pathways for the migration of photogenerated electrons, thus facilitate the electron holes separation, and these lead to the longer lifetime of the photogenerated electron–holes.^45,54^ Fe doping generates additional band structure states due to its unpaired d-electrons. These modifications lower the band gap to values as low as 2.93 eV for Fe-UiO-66 and 2.37 eV for Fe-UiO-66-(NH_2_), facilitating efficient electron excitation under visible light. The combined effects of NH_2_ functionalization and metal doping result in an improved photocatalytic degradation, underscoring the synergy between these structural modifications. To the best of our knowledge, no studies have reported the use of MOFs for the photodegradation of 1-NA. In fact, only a limited number of reports specifically address the photodegradation or photoadvanced oxidation of 1-NA. Table S4 provides a comparative summary of the materials previously investigated for photobased degradation of 1-NA, along with the corresponding experimental conditions employed in each study. In the following section, experiments will be conducted with the best performing catalysts, 15%(Ti–Zr)-UiO-66-(NH_2_) and Fe-UiO-66-(NH_2_).

### Free Radical Capture Experiments

The reactive species involved in the photoassisted oxidation of 1-NA may include superoxide radicals, hydroxyl radicals, electrons, and/or holes. To investigate the role of these species in the degradation of 1-NA using 15%(Ti–Zr)-UiO-66-(NH_2_) and Fe-UiO-66-(NH_2_) as photocatalysts, scavenging experiments are performed. Different scavengers are employed: benzoic acid (BA) for superoxide radicals (O_2_^·–^), 2-propanol for hydroxyl radicals (OH^·^), AgNO_3_ for electrons (e^^–^^), and triethylamine (TEA) for holes (h^+^). The catalytic results, in the presence of the scavengers, were presented in Figure 7a,b.

**Figure 7:**
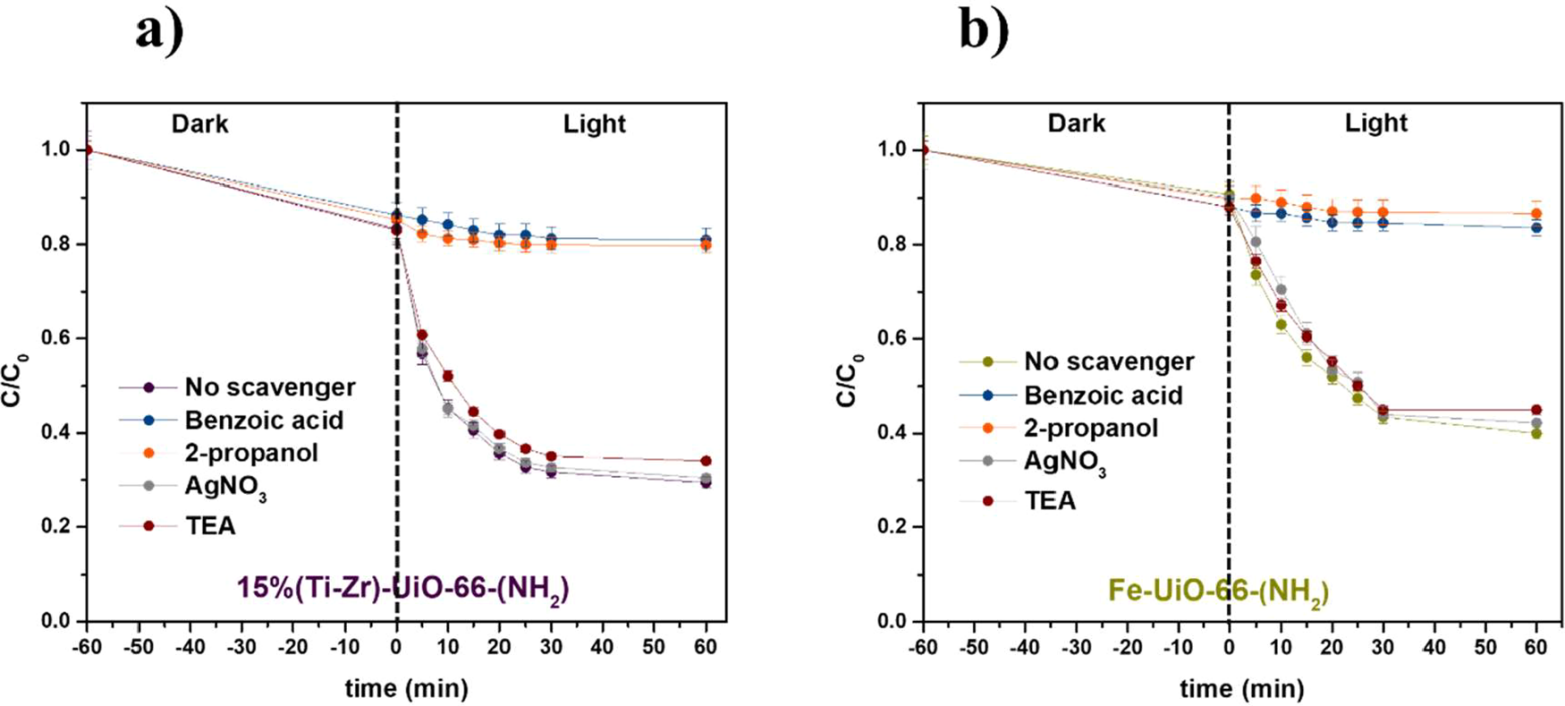
Effect of different scavengers on the photocatalytic degradation of 1-NA with (a) 15%(Ti–Zr)-UiO-66-(NH_2_) and (b) Fe-UiO-66-(NH_2_).

In the case of 15%(Ti–Zr)-UiO-66-NH_2_, the inhibitory effects of TEA and AgNO_3_ are minimal, indicating that holes (h^+^) and direct electron (e^–^) interactions play a negligible role in the photocatalytic degradation of 1-NA. However, significant suppression of activity is observed with the addition of 2-propanol and BA. The degradation efficiency drops from about 75% (no scavenger) to ∼20% with 2-propanol and ∼18% with BA, suggesting that hydroxyl radicals (OH^·^) and superoxide radicals (O_2_^·–^) are the primary reactive species involved in the degradation process. A mechanism can be deduced. Upon irradiation, an electron is transferred from the excited H_2_BDC-NH_2_ ligand to the Ti–O–Zr-oxo-clusters, facilitating a ligand-to-cluster charge transfer (LCCT). The incorporated Ti^4^^+^ ions within the MOF framework are subsequently reduced to Ti^3^^+^ by photoexcited electrons (eqs 2 and 3).^28,48^ The resulting Ti^3^^+^ species then react with O_2_, leading to the formation of superoxide radicals (O_2_^·–^) (eq 4). Importantly, the Ti^4^^+^/Ti^3^^+^ redox cycle plays a key role in enhancing charge transfer within the MOF, thereby improving the photocatalytic efficiency. Meanwhile, the photoinduced holes (h^+^) remaining in the HOMO interact with hydroxyl groups from surface-adsorbed water (eq 9), generating highly reactive hydroxyl radicals (OH^·^), which contribute to the oxidation process.

Similarly, for Fe-UiO-66-(NH_2_), the inhibitory effects of 2-propanol and BA confirm that hydroxyl and superoxide radicals are key intermediates in the photocatalytic degradation of 1-NA (Figure 7b). The degradation efficiency decreases from around 65% without scavengers to about 22% with 2-propanol and 18% with BA. For Fe-doped UiO-66-(NH_2_), under irradiation, two possible pathways can occur. For the first pathway (i), excited electrons from H_2_BDC-NH_2_ can be transferred to FeO_*x*_ via a LCCT (eq 5). These electrons then migrate along the oxygen bridge to the Zr-oxo cluster, resulting in a charge-separated state. The photogenerated electrons subsequently react with O_2_, generating superoxide radicals (O_2_^·–^). For the second pathway (ii), electrons from the 3d states of Fe can be directly excited, initiating a MCCT (eq 6). These electrons migrate along the oxygen bridge to the Zr-oxo cluster, leading to a charge-separated state. The photogenerated electrons react with O_2_, forming superoxide radicals (O_2_^·–^) (eq 7), while the corresponding holes oxidize water to produce hydroxyl radicals (OH^·^) (eq 9). In Ti- and Fe-doped UiO-66-(NH_2_), the LCCT and MCCT mechanisms effectively suppress electron–hole recombination, thereby enhancing the photocatalytic degradation efficiency under visible light irradiation.

The proposed mechanism is described below:

$$(\mathrm{Ti}-\mathrm{Zr})‐\mathrm{UiO}‐66‐({\mathrm{NH}}_{2})+\mathrm{h\nu}\to e^{-}+h^{+}$$ 2

$${\mathrm{Ti}}^{4+}+e^{-}\to {\mathrm{Ti}}^{3+}$$ 3

$${\mathrm{Ti}}^{3+}+O_{2}\to {O_{2}}^{\cdot -}$$ 4

$$\mathrm{Fe}-\mathrm{UiO}‐66‐({\mathrm{NH}}_{2})+\mathrm{h\nu}\to e^{-}+h^{+}$$ 5

$${\mathrm{Fe}}^{\mathrm{III}}-\mathrm{UiO}‐66‐({\mathrm{NH}}_{2})+\mathrm{h\nu}\to {\mathrm{Fe}}^{\mathrm{IV}}-\mathrm{UiO}‐66‐({\mathrm{NH}}_{2})e^{-}+h^{+}$$ 6

$${\mathrm{Fe}}^{\mathrm{IV}}-\mathrm{UiO}‐66‐({\mathrm{NH}}_{2})e^{-}+O_{2}\to {O_{2}}^{\cdot -}$$ 7

$${O_{2}}^{\cdot -}+1‐\mathrm{Naphthylamine}\to \mathrm{degradation}\;\mathrm{product}$$ 8

$$h^{+}+{\mathrm{OH}}^{-}/H_{2}O\to {\mathrm{OH}}^{\cdot }$$ 9

$${\mathrm{OH}}^{\cdot }+1‐\mathrm{Naphthylamine}\to \mathrm{degradation}\;\mathrm{product}$$ 10

### Effect of 1-NA Concentration and Catalyst Dosage

To test the performance of 15%(Ti–Zr) and Fe-UiO-66-(NH_2_) in the degradation of 1-NA at different initial concentrations, experiments were conducted with concentrations ranging from 5 to 15 ppm (Figure S6a,b). The results indicate that as the initial concentration of 1-NA increases, the degradation efficiency improves up to 90% in 15%(Ti–Zr)- UiO-66-(NH_2_) and 80% in Fe-UiO-66-(NH_2_); however, at 15 ppm, the efficiency begins to decline. For concentrations below 15 ppm, the amount of 1-NA degraded increases, suggesting that the degradation process is more effective at lower concentrations. Nevertheless, since the solubility limit of 1-NA in water is approximately 20 mg/L,^57^ the observed decline at 15 ppm may be attributed to the fact that the system approaches saturation, and the applied conditions are no longer sufficient to effectively degrade 1-NA at or near its solubility limit.

As shown in Figure S6c,d, increasing the catalyst dosage enhances the degradation of 1-NA from 75 to 80% in 15%(Ti–Zr)- UiO-66-(NH_2_), and from 65 to 70% in Fe-UiO-66-(NH_2_). This improvement is attributed to the generation of more electrons as the catalyst amount rises. Consequently, the Ti^4^^+^ and Fe^3^^+^ species capture more photogenerated electrons, leading to the formation of additional superoxide radicals. However, an excessive catalyst dosage can result in reduced photodegradation efficiency. This decrease is likely due to visible light loss caused by scattering effects, as well as the aggregation of catalyst particles and the resulting turbidity. These factors reduce photon absorption, thereby reducing the degradation efficiency.

A similar trend was observed in the photodegradation of methyl orange using mesoporous titania: at lower initial pollutant concentrations, complete degradation was achieved within 180 min of irradiation. Additionally, the degradation efficiency increased with TiO_2_ content up to 0.5 g/L, beyond which it remained constant. However, the addition of higher quantities of photocatalyst would have no effect on the degradation efficiency.^58^

### Effect of pH

Figure 8 illustrates the influence of the pH on the photocatalytic efficacy of catalysts. The initial solution pH, without any adjustment, was measured to be 6.5. When the pH was increased from 2 to 6.5, a significant enhancement from 22 to 75%, and from 10 to 65% in the degradation efficiency of 1-NA was observed for 15%(Ti–Zr)-UiO-66-(NH_2_) and Fe-UiO-66-(NH_2_), respectively. However, further increasing the pH to 11 resulted in a decline in the degradation performance. MOFs such as the evaluated Ti- and Fe-doped-UiO-66-(NH_2_) are crystalline porous materials constructed via coordination bonding between metal clusters (e.g., Ti, Zr, Fe) and organic ligands. Under strongly acidic conditions, partial leaching of Ti and Fe ions from the MOF framework may occur, leading to structural instability or collapse^59,60^ Additionally, the presence of hydrogen ions in the acidic medium can neutralize OH^·^ radicals, leading to a reduction in the overall concentration of free radicals in the system.^61^ Also, in an acidic medium (pH < 5), the scavenging effect of protons on O_2_^·–^ (the primary species responsible for 1-NA oxidation, as illustrated in Figure 7), leads to the formation of hydroperoxide radicals (HO_2_^·^), which have a lower oxidative potential than O_2_^·–^.^62^ Conversely, in highly alkaline environments, hydroxide species (e.g., Ti­(OH)_4_, Fe­(OH)_3_) can form,^63,64^ potentially disrupting the crystalline structure. A comparable trend was observed for the PMS/Fe-UiO-66 system, where the degradation rate of sulfameter increased as the pH value increased from 1 to 6.3. However, a further increase in pH up to 11 led to a decline in the degradation efficiency.^54^

**Figure 8:**
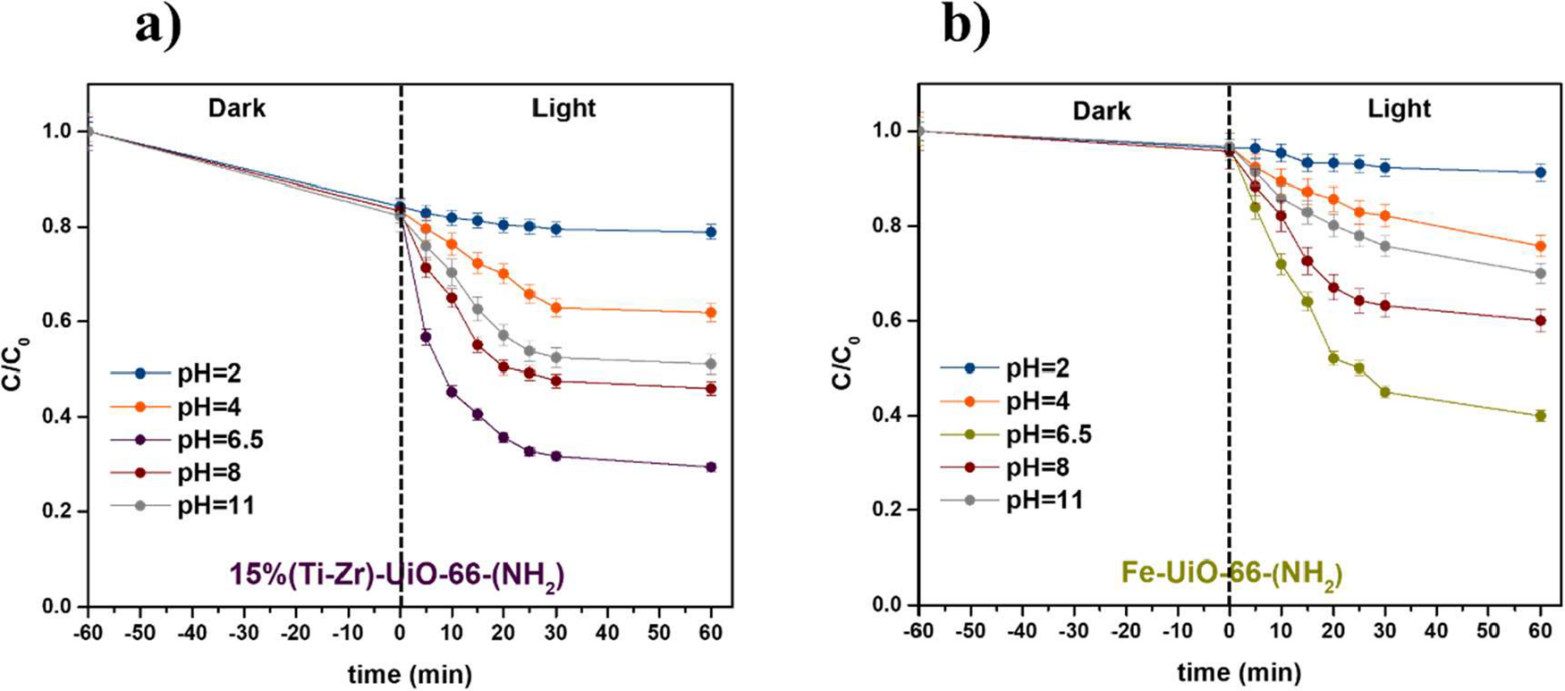
Degradation of 1-NA at different pH with (a) 15%(Ti–Zr)-UiO-66-(NH_2_) and (b) Fe-UiO-66-(NH_2_).

1-NA exists in different ionic forms depending on the pH of the solution. At low pH (<5), 1-NA is predominantly in its protonated form (1-NAH^+^), making it more soluble in water but less reactive toward oxidation by superoxide radicals (O_2_^·–^). As pH increases toward neutral conditions,1-NA is mostly in its neutral form, which facilitates interaction with photocatalyst active sites and improves degradation efficiency. The distribution of these species as a function of pH for 1-NA is illustrated in Figure S7.

### Proposed Degradation Intermediates

A noticeable change in the reaction mixture was observed during the photocatalytic degradation, transitioning from colorless to pigmented following the degradation of 1-NA. The reactive species (OH^·^ and O_2_^·–^) are capable of oxidizing the probe compound, leading to the formation of yellowish intermediates. The intensity of this yellow coloration increased progressively over time, indicating a continuous transformation of the degradation products (Figure S8).

To establish a correlation between pigment formation and degradation mechanism, UV–vis spectrometry was performed. As shown in Figure S9, the maximum absorbance (λ_max_) of 1-NA alone was recorded at 308 nm. Following photodegradation, the emergence of a shoulder peak at 407 nm suggests the formation of juglone according to the literature,^65^ a known intermediate in the photodegradation pathway of 1-NA, as reported in previous studies.^10^ The reduction in absorbance at λ_max_ corresponds to the decline in the 1-NA concentration, supporting the conversion of the probe compound into less persistent degradation products. Additionally, another naphthoquinone, identified as lawsone, was detected, as confirmed by the appearance of an additional peak in the HPLC chromatogram (Figures S10 and S11) at a retention time of 3.08 min. This finding, consistent with previous reports,^66^ provides further evidence of 1-NA photodegradation rather than a mere decrease in concentration over time.

Based on these findings and previous works,^10,64^ a potential photodegradation mechanism can be proposed. Hydroxyl radicals (OH^·^) are highly reactive electrophilic species capable of degrading organic compounds through hydrogen abstraction or radical addition.^67^ Superoxide anion radicals (O_2_^·–^,) exhibit a moderate oxidative potential relative to hydroxyl radicals (OH^·^) and display a greater selectivity in their reactions. While not highly reactive on their own, O_2_^·–^ can indirectly facilitate the degradation of organic pollutants by participating in redox reactions, including hydrogen abstraction and oxidation reactions.^68^ The oxidation of amino groups can yield nitroso functionalities, which are further oxidized to nitro compounds. In the case of 1-NA, its dehydrogenation by OH^·^ and/or O_2_^·–^ results in compound {1}, which undergoes subsequent oxidation by OH^·^ to produce compound {3}. Due to the high reactivity of OH^·^, it predominantly targets the ortho or para positions of aromatic rings. This reactivity enables compound {1} to form compounds {2} and {4} through OH addition. Furthermore, nucleophilic aromatic substitution facilitated by OH^·^ leads to the formation of compounds {5} and {6} by direct substitution of the nitroso group. These intermediates, compounds {5} and {6}, can undergo oxidation via oxidative ring cleavage mediated by O_2_^·–^, yielding compounds {7} and {8}. Similarly, compounds {5} and {6} are initially reduced to hydroxyl ion derivatives {9} and {10} before further reduction to naphthoquinones {11} and {12}. This redox cycling promotes degradation of target contaminants. Oxidation of compounds {11} and {12} through OH addition forms lawsones {13, 16} and juglones {14, 17}. The yellowish coloration observed in the reaction medium (Figure S8) confirms the presence of these compounds, consistent with findings from previous studies.^64,69,70^ Further oxidation of naphthoquinones {11} and {12} by O_2_^·–^ results in the formation of anhydride compound {15}, which subsequently leads to phthalic acid {18}. This observation aligns with prior research.^71^ Decarboxylation of phthalic acid {18} generates benzoic acid {19}. The complete oxidation or mineralization of benzoic acid leads to the breakdown of the aromatic ring, resulting in the formation of carbon dioxide and water (Scheme S1).

### Recycling Tests

To evaluate the reusability of 15%(Ti–Zr)-UiO-66-(NH_2_) and Fe-UiO-66-(NH_2_), recyclability tests were conducted by washing the MOF crystals after each photodegradation cycle. The photocatalytic experiment was repeated four additional times. Figure 9 highlights that the 1-NA degradation efficiency remained good over five-cycles for the 2 MOFs. The MOFs exhibited consistent performance throughout the cycles with no significant decline in degradation efficiency during the first four cycles. This indicates that these MOFs maintain a high degradation capacity, demonstrating the long-term stability of 15%(Ti–Zr)-UiO-66-(NH_2_) and Fe-UiO-66-(NH_2_). Consequently, these materials show strong potential as recyclable catalysts for the removal of micropollutants.

**Figure 9:**
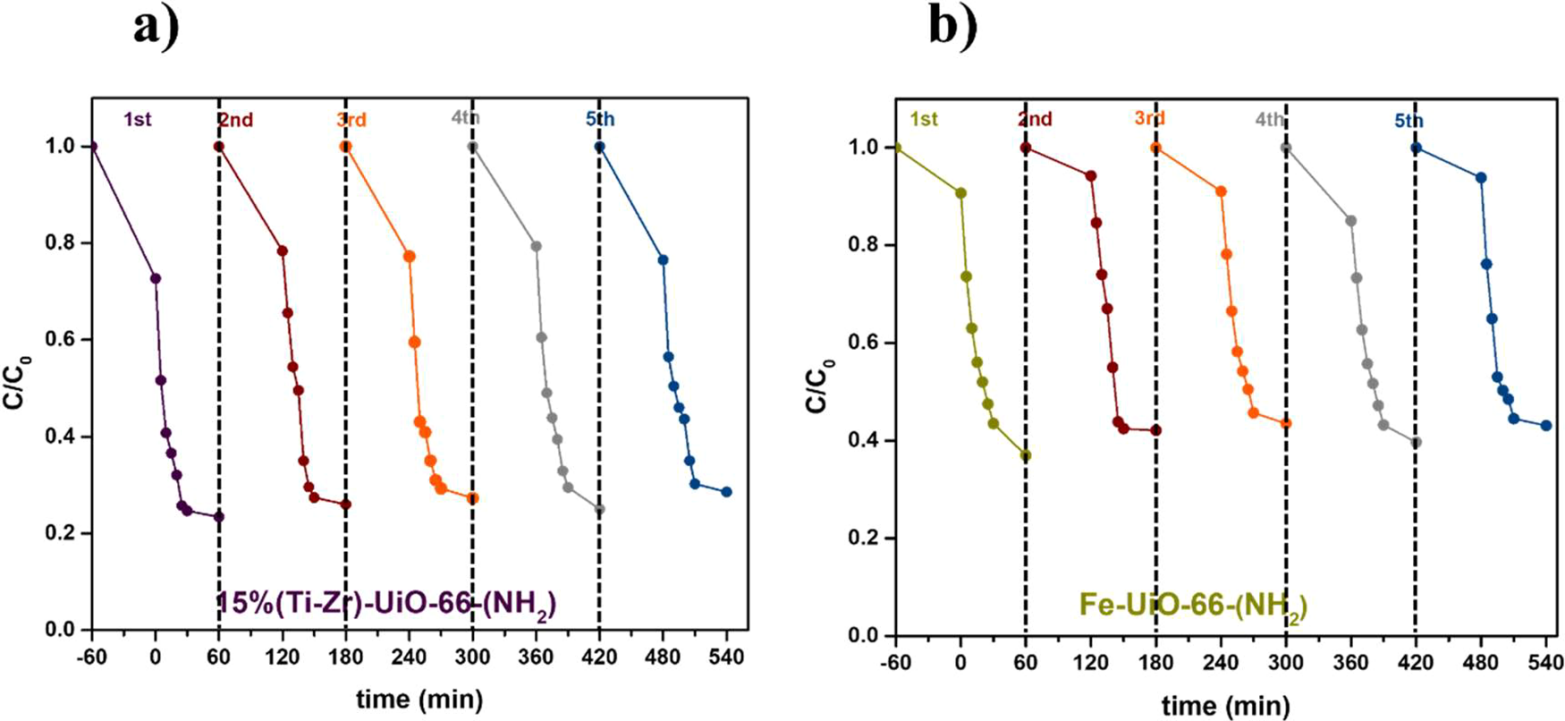
Degradation of 1-NA over five-cycles using (a) 15%(Ti–Zr)-UiO-66-(NH_2_) and (b) Fe-UiO-66-(NH_2_). Experimental conditions: [1-NA]_o_ = 5 mg L^–1^; *m*_catalyst_ = 35 mg; and light intensity = 100 W.

We conducted PXRD and SEM analyses on regenerated samples of 15% (Ti–Zr)-UiO-66-(NH_2_) and Fe-UiO-66-(NH_2_). The results show that both 15% (Ti–Zr)-UiO-66-(NH_2_) and Fe-UiO-66-(NH_2_) remain stable after reuse. The SEM images (Figure S12) confirm that the shape of the materials does not change and the PXRD patterns (Figure S13) before and after photodegradation are almost the same. This means that the materials maintain their crystallinity and can be reused effectively.

## Conclusions

The activity of UiO-66-based photocatalysts for 1-NA degradation is significantly influenced by the presence of a functional group in the organic linkers and the metal doping in the clusters of the MOF. Solvothermal and microwave-assisted methods were successfully employed to synthesize Ti- and Fe-doped UiO-66-(NH_2_) based MOFs. The enhanced photocatalytic performance of these materials can be attributed to the introduction of an amino group, which modified the electronic structure of UiO-66 and reduced its band gap. Additionally, titanium acted as an electron mediator through the formation of oxo-bridged hetero-Zr–Ti clusters, while iron facilitated charge transfer from the metal to the cluster, significantly improving the optical properties compared with the original UiO-66. Under visible light irradiation, the degradation of 1-NA reached 75 and 65% within less than 60 min for (Ti–Zr)-UiO-66-(NH_2_) and Fe-UiO-66-(NH_2_), respectively. This improved performance can be ascribed to the synergistic effects of enhanced light absorption and efficient separation of the photogenerated electron–hole pairs. Furthermore, increasing the amount of doped metal led to a higher degradation efficiency. The photocatalytic degradation of 1-NA was driven primarily by hydroxyl and superoxide radicals (OH^·^ and O_2_^·–^), which acted as the main reactive species. These findings provide valuable insights into the design and application of MOFs for the treatment of micropollutants, offering a promising pathway for the development of effective and sustainable photocatalytic materials. Future work will focus on extending the application of the synthesized MOFs to the degradation of a broader range of micropollutants, including those present in real river water. Additionally, further exploration of metal doping and functionalization with various groups may not only enhance the degradation efficiency for other contaminants but also open new avenues for catalytic applications beyond water treatment.

## Supplementary Material



## Notes

Materials used for synthesis; photographs of samples; characterization of additional results; FT-IR spectra of the MOFs samples; XPS spectra of C 1s, O 1s, and N 1s; atomic percentages of 15%(Ti–Zr)-UiO-66-(NH_2_) and Fe-UiO-66-(NH_2_); quantitative results obtained during the characterization of the MOFs; values of the band gap energy (*E*_g_) of the MOFs; additional photodegradation results; scheme of the reactor; kinetics data; 1-NA species distribution; UV–vis spectra; HPLC chromatograms; effect of 1-NA and catalyst dosage; picture of the pigmented solution; proposed mechanism of 1-NA degradation; post-photodegradation additional results; and SEM images and PXRD patterns of regenerated MOFs (PDF)

## References

[1] Huang L., Lv Y., Wu S., Liu P., Xiong W., Hao F. (2019). Activated carbon supported bimetallic catalysts with combined catalytic
effects for aromatic nitro compounds hydrogenation under mild conditions. Appl. Catal. A Gen.

[2] Yu C., Fu J., Muzzio M., Shen T., Su D., Zhu J. (2017). CuNi Nanoparticles Assembled on Graphene for Catalytic
Methanolysis
of Ammonia Borane and Hydrogenation of Nitro/Nitrile Compounds. Chem. Mater..

[3] Mei B., Wang Y., Zhou J., Yang X., Yu Y., Xu L. (2024). Experimental and simulation study on the solubility
of 1-naphthylamine in twelve neat solvents. J. Mol. Liq..

[4] Guzmán
Mar J. L., López Martínez L., López de Alba J. E., Castrejón Durán J. E., Cerdà Martín V. (2006). Optical fiber reflectance sensor
coupled to a multisyringe flow injection system for preconcentration
and determination of 1-naphthylamine in water samples. Anal. Chim. Acta.

[5] Sekar, N. Acid dyes. In Handbook of Textile and Industrial Dyeing: Principles, Processes and Types of Dyes; Woodhead Publishing Limited, 2011; Vol. 1, pp 486–514.

[6] Gao J., Wei X., Yang W., Lv D., Qu J., Chen H. (2007). Determination of 1-naphthylamine by using oscillating chemical reaction. J. Hazard Mater..

[7] Edebali Ö, Krupčíková S., Goellner A., Vrana B., Muz M., Melymuk L. (2024). Tracking Aromatic
Amines from Sources to Surface Waters. Environ.
Sci. Technol. Lett..

[8] Kim
Man M. M. (2021). Glove Industry Spikes during Covid-19 Pandemic: A Case Study of Comfort
Gloves Berhad (CGB). Int. Bus Res..

[9] Haidar O., Roques-Carmes T., Gouda A., Tabaja N., Toufaily J., Hmadeh M. (2024). Defect-Rich
Metal-Organic Framework Nanocrystals for
Removal of Micropollutants from Water. ACS Appl.
Nano Mater..

[10] Liu J., So H. L., Chu W. (2022). Degradation of 1-naphthylamine by
a UV enhanced Fe^2+^/peroxymonosulfate system: A novel pH-dependent
activation pathway. Chem. Eng. J..

[11] Dong H., Zeng G., Tang L., Fan C., Zhang C., He X. (2015). An overview on limitations
of TiO_2_-based
particles for photocatalytic degradation of organic pollutants and
the corresponding countermeasures. Water Res..

[12] Zimny K., Roques-Carmes T., Carteret C., Stébé M.
J., Blin J. L. (2012). Synthesis
and photoactivity of ordered mesoporous titania
with a semicrystalline framework. J. Phys. Chem.
C.

[13] Gao Y., Xia J., Liu D., Kang R., Yu G., Deng S. (2019). Synthesis
of mixed-linker Zr-MOFs for emerging contaminant adsorption and photodegradation
under visible light. Chem. Eng. J..

[14] Jrad A., Al Sabeh G., Hannouche K., Al Natour R., Haidar O., Sammoury H. (2023). Critical
Role of Defects
in UiO-66 Nanocrystals for Catalysis and Water Remediation. ACS Appl. Nano Mater..

[15] Damacet P., Hannouche K., Gouda A., Hmadeh M. (2025). Controlled Growth of
Highly Defected Zirconium-Metal-Organic Frameworks via a Reaction-Diffusion
System for Water Remediation. ACS Appl. Mater.
Interfaces.

[16] He L., Dong Y., Zheng Y., Jia Q., Shan S., Zhang Y. (2019). A novel magnetic
MIL-101­(Fe)/TiO_2_ composite for photo
degradation of tetracycline under solar light. J. Hazard Mater..

[17] Li N., Liu X., Zhou J., Chen W., Liu M. (2020). Encapsulating
CuO quantum
dots in MIL-125­(Ti) coupled with g-C3N4 for efficient photocatalytic
CO_2_ reduction. Chem. Eng. J..

[18] Ali-Ahmad A., Hamieh T., Roques-Carmes T., Hmadeh M., Toufaily J. (2024). Effect of
amino functional groups on the surface properties and Lewis’s
acid base parameters of UiO-66­(NH_2_) by inverse gas chromatography. Heliyon.

[19] Issa R., Ibrahim F. A., Al-Ghoul M., Hmadeh M. (2021). Controlled
growth and
composition of multivariate metal-organic frameworks-199 via a reaction-diffusion
process. Nano Res..

[20] Wang R., Gu L., Zhou J., Liu X., Teng F., Li C. (2015). Quasi-Polymeric Metal-Organic
Framework UiO-66/g-C_3_N_4_ Heterojunctions for
Enhanced Photocatalytic Hydrogen Evolution
under Visible Light Irradiation. Adv. Mater.
Interfaces.

[21] Ali
Akbar Razavi S., Morsali A. (2019). Linker functionalized metal-organic
frameworks. Coord. Chem. Rev..

[22] Bedia J., Muelas-Ramos V., Peñas-Garzón M., Gómez-Avilés A., Rodríguez J. J., Belver C. (2019). A review on the synthesis and characterization
of metal organic frameworks for photocatalytic water purification. Catalysts.

[23] Gómez-Avilés A., Muelas-Ramos V., Bedia J., Rodriguez J. J., Belver C. (2020). Thermal post-treatments
to enhance the water stability
of NH_2_-MIL-125­(Ti). Catalysts.

[24] Liu X., Demir N. K., Wu Z., Li K. (2015). Highly Water-Stable
Zirconium Metal-Organic Framework UiO-66 Membranes Supported on Alumina
Hollow Fibers for Desalination. J. Am. Chem.
Soc..

[25] Zhang Y., Zhou J., Feng Q., Chen X., Hu Z. (2018). Visible light
photocatalytic degradation of MB using UiO-66/g-C_3_N_4_ heterojunction nanocatalyst. Chemosphere.

[26] Chen T. F., Han S. Y., Wang Z. P., Gao H., Wang L. Y., Deng Y. H. (2019). Modified UiO-66 frameworks
with methylthio, thiol and
sulfonic acid function groups: The structure and visible-light-driven
photocatalytic property study. Appl. Catal.
B Environ.

[27] Mu X., Jiang J., Chao F., Lou Y., Chen J. (2018). Ligand modification
of UiO-66 with an unusual visible light photocatalytic behavior for
RhB degradation. Dalt Trans.

[28] Sun D., Liu W., Qiu M., Zhang Y., Li Z. (2015). Introduction of a mediator
for enhancing photocatalytic performance via post-synthetic metal
exchange in metal-organic frameworks (MOFs). Chem. Commun..

[29] Panda J., Singha D., Panda P. K., Chandra Tripathy B., Rana M. K. (2022). Experimental and DFT Study of Transition Metal Doping
in a Zn-BDC MOF to Improve Electrical and Visible Light Absorption
Properties. J. Phys. Chem. C.

[30] Chen S., Hai G., Gao H., Chen X., Li A., Zhang X. (2021). Modulation
of the charge transfer behavior of Ni­(II)-doped NH_2_-MIL-125­(Ti):
Regulation of Ni ions content and enhanced photocatalytic
CO_2_ reduction performance. Chem.
Eng. J..

[31] Tu J., Zeng X., Xu F., Wu X., Tian Y., Hou X. (2017). Microwave-induced
fast incorporation of titanium into
UiO-66 metal-organic frameworks for enhanced photocatalytic properties. Chem. Commun..

[32] Zhu R., Cai M., Fu T., Yin D., Peng H., Liao S. (2023). Fe-Based Metal Organic Frameworks (Fe-MOFs) for Bio-Related Applications. Pharmaceutics.

[33] Cai J., Peng Y., Jiang Y., Li L., Wang H., Li K. (2023). Application of Fe-MOFs in Photodegradation
and Removal of Air and
Water Pollutants: A Review. Molecules.

[34] Xu C., Pan Y., Wan G., Liu H., Wang L., Zhou H. (2019). Turning on Visible-Light Photocatalytic C-H Oxidation
over Metal-Organic
Frameworks by Introducing Metal-to-Cluster Charge Transfer. J. Am. Chem. Soc..

[35] Chen X., Kuwahara Y., Mori K., Louis C., Yamashita H. (2020). A hydrophobic
titanium doped zirconium-based metal organic framework for photocatalytic
hydrogen peroxide production in a two-phase system. J. Mater. Chem. A.

[36] Saliba D., El Jamal S. E., Jonderian A., Ammar M., Hmadeh M., Al-Ghoul M. (2020). Tuning the structural properties of cadmium-Aluminum
layered double hydroxide for enhanced photocatalytic dye degradation. RSC Adv..

[37] Minh
Nguyet Bui T., Ky Vo T., Hoang Yen Phuong N., Hung Nguyen V., Cuong Nguyen V., Hung Nguyen Q. (2025). Fe­(III)-incorporated UiO-66­(Zr)–NH_2_ frameworks:
Microwave-assisted scalable production and their enhanced photo-Fenton
degradation catalytic activities. Sep Purif
Technol..

[38] Xu W., Dong M., Di L., Zhang X. (2019). A facile method for
preparing uio-66 encapsulated ru catalyst and its application in plasma-assisted
CO_2_ methanation. Nanomaterials.

[39] Kim H. G., Choi K., Lee K., Lee S., Jung K. W., Choi J. W. (2021). Controlling the structural robustness of zirconium-based
metal organic frameworks for efficient adsorption on tetracycline
antibiotics. Water.

[40] Patel S. B., Baker N., Marques I., Hamlekhan A., Mathew M. T., Takoudis C. (2017). Transparent TiO_2_ nanotubes on zirconia for biomedical applications. RSC Adv..

[41] Hirata T., Kitajima M., Nakamura K. G., Asari E. (1994). Infrared and Raman
Spectra of Solid Solutions Ti_1–x_Zr_x_O_2_ (x⩽0.1). J. Phys. Chem. Solids.

[42] Wu X., Lv C., Yu S., Li M., Ye J., Zhang X. (2020). Uranium (VI) removal
from aqueous solution using iron-carbon micro-electrolysis
packing. Sep Purif Technol..

[43] Du X., Yi X., Wang P., Deng J., Wang C. C. (2019). Enhanced photocatalytic
Cr­(VI) reduction and diclofenac sodium degradation under simulated
sunlight irradiation over MIL-100­(Fe)/g-C_3_N_4_ heterojunctions. Cuihua Xuebao/Chinese J.
Catal.

[44] Mukhopadhyay S., Shimoni R., Liberman I., Ifraemov R., Rozenberg I., Hod I. (2021). Assembly of a Metal–Organic Framework (MOF) Membrane on a
Solid Electrocatalyst: Introducing Molecular-Level Control Over Heterogeneous
CO_2_ Reduction. Angew. Chem..

[45] Wang A., Zhou Y., Wang Z., Chen M., Sun L., Liu X. (2016). Titanium incorporated
with UiO-66­(Zr)-type Metal-Organic Framework
(MOF) for photocatalytic application. RSC Adv..

[46] Jrad A., Hmadeh M., Awada G., Chakleh R., Ahmad M. (2021). Efficient
biofuel production by MTV-UiO-66 based catalysts. Chem. Eng. J..

[47] Sofi F. A., Majid K., Mehraj O. (2018). The visible light driven
copper based
metal-organic-framework heterojunction:HKUST-1@Ag-Ag_3_PO_4_ for plasmon enhanced visible light photocatalysis. J. Alloys Compd..

[48] Santiago
Portillo A., Baldoví H. G., García Fernandez M. T., Navalón S., Atienzar P., Ferrer B. (2017). Ti as
Mediator in the Photoinduced Electron Transfer of Mixed-Metal NH_2_-UiO-66­(Zr/Ti): Transient Absorption Spectroscopy Study and
Application in Photovoltaic Cell. J. Phys. Chem.
C.

[49] Kalaj M., Momeni M. R., Bentz K. C., Barcus K. S., Palomba J. M., Paesani F. (2019). Halogen
bonding in UiO-66 frameworks promotes superior
chemical warfare agent simulant degradation. Chem. Commun..

[50] Shen L., Liang R., Luo M., Jing F., Wu L. (2015). Electronic
effects of ligand substitution on metal-organic framework photocatalysts:
The case study of UiO-66. Phys. Chem. Chem.
Phys..

[51] Wang Y. L., Zhang S., Zhao Y. F., Bedia J., Rodriguez J. J., Belver C. (2021). UiO-66-based metal
organic frameworks for the photodegradation
of acetaminophen under simulated solar irradiation. J. Environ. Chem. Eng..

[52] Lammert M., Wharmby M. T., Smolders S., Bueken B., Lieb A., Lomachenko K. A. (2015). Cerium-based metal organic
frameworks with
UiO-66 architecture: Synthesis, properties and redox catalytic activity. Chem. Commun..

[53] Lv S. W., Liu J. M., Zhao N., Li C. Y., Wang Z. H., Wang S. (2020). A novel cobalt doped MOF-based photocatalyst with great applicability
as an efficient mediator of peroxydisulfate activation for enhanced
degradation of organic pollutants. New J. Chem..

[54] Lin Y., Zhang Y., Li G. (2022). Promotion
of sulfameter degradation
by coupling persulfate and photocatalytic advanced oxidation processes
with Fe-doped MOFs. Sep Purif Technol..

[55] Feng Y., Chen Q., Cao M., Ling N., Yao J. (2019). Defect-Tailoring
and Titanium Substitution in Metal – Organic Framework UiO-66-NH_2_ for the Photocatalytic Degradation of Cr­(VI) to Cr­(III). ACS Appl. Nano Mater..

[56] Cao J., Yang Z.-h., Xiong W.-p., Zhou Y.-y., Peng Y.-r., Li X. (2018). One-step synthesis of Co-doped UiO-66 nanoparticle
with enhanced removal efficiency of tetracycline: Simultaneous adsorption
and photocatalysis. Chem. Eng. J..

[57] Xu W., Liu X., Cai J., Xue T., Tang K. (2022). Synthesis of reusable
cyclodextrin polymers for removal of naphthol and naphthylamine from
water. Environ. Sci. Pollut Res..

[58] Blin J. L., Stébé M. J., Roques-Carmes T. (2012). Use of ordered
mesoporous titania with semi-crystalline framework as photocatalyst. Colloids Surfaces A Physicochem Eng. Asp.

[59] Rojas S., García-González J., Salcedo-Abraira P., Rincón I., Castells-Gil J., Padial N. M. (2022). Ti-based
robust MOFs in the combined photocatalytic degradation of emerging
organic contaminants. Sci. Rep..

[60] Gao Y., Li S., Li Y., Yao L., Zhang H. (2017). Accelerated
photocatalytic
degradation of organic pollutant over metal-organic framework MIL-53­(Fe)
under visible LED light mediated by persulfate. Appl. Catal. B Environ.

[61] Huang Y. H., Huang Y. F., Huang C., Chen C. Y. (2009). Efficient
decolorization
of azo dye Reactive Black B involving aromatic fragment degradation
in buffered Co^2+^/PMS oxidative processes with a ppb level
dosage of Co^2+^-catalyst. J. Hazard
Mater..

[62] Brillas E., Sirés I., Oturan M. A. (2009). Electro-fenton process and related
electrochemical technologies based on fenton’s reaction chemistry. Chem. Rev..

[63] Zhu J., Li P. Z., Guo W., Zhao Y., Zou R. (2018). Titanium-based
metal–organic frameworks for photocatalytic applications. Coord. Chem. Rev..

[64] So H. L., Chu W., Wang Y. H. (2019). Naphthalene degradation
by Fe^2+^/Oxone/UV
– Applying an unconventional kinetics model and studying the
reaction mechanism. Chemosphere.

[65] Ebrahimi I., Parvinzadeh Gashti M. (2015). Extraction
of juglone from Pterocarya fraxinifolia
leaves for dyeing, anti-fungal finishing, and solar UV protection
of wool. Color Technol..

[66] Babula P., Mikelova R., Adam V., Kizek R., Havel L., Sladky Z. (2006). Using of liquid chromatography
coupled with diode array
detector for determination of naphthoquinones in plants and for investigation
of influence of pH of cultivation medium on content of plumbagin in
Dionaea muscipula. J. Chromatogr B Anal Technol.
Biomed Life Sci..

[67] Lee J., Von Gunten U., Kim J. H. (2020). Persulfate-Based Advanced Oxidation:
Critical Assessment of Opportunities and Roadblocks. Environ. Sci. Technol..

[68] Otálora A., Lerma T. A., Arrieta-Urango Y., Palencia M. (2021). Emerging organic pollutants
in aqueous environments: Detection, monitoring, and removal techniques. J. Sci. with Technol. Appl..

[69] Hijji Y. M., Barare B., Zhang Y. (2012). Lawsone (2-hydroxy-1,4-naphthoquinone)
as a sensitive cyanide and acetate sensor. Sensors
Actuators, B Chem..

[70] Zaheer Z., Danish E. Y., Kosa S. A. (2021). 2-Hydroxy-1, 4-napthoquinone
solubilization,
thermodynamics and adsorption kinetics with surfactant. Chinese J. Chem. Eng..

[71] Rodriguez S., Vasquez L., Costa D., Romero A., Santos A. (2014). Oxidation
of Orange G by persulfate activated by Fe­(II), Fe­(III) and zero valent
iron (ZVI). Chemosphere.

