# Supplementary material for: Cation-Doped Amino-Functionalized Zirconium MOF Nanocrystals for Enhanced Photocatalytic Degradation of 1‑Naphthylamine
Source: Langmuir. 2025 Dec 26;42(1):1490–503. doi: 10.1021/acs.langmuir.5c05475 (PMC12810380; doi:10.1021/acs.langmuir.5c05475)
Supplement: Supplementary file 1 [file la5c05475_si_001.pdf]

Supporting Information

Cation-Doped Amino-Functionalized Zirconium MOF Nanocrystals for Enhanced Photocatalytic Degradation of 1-Naphthylamine

Ola Haidar[1,2,3,4], Hassan Wehbe[1], Thibault Roques-Carmes[2*], Joumana Toufaily[3,4*], Mohamad Hmadeh[1*]

[1] Department of Chemistry, American University of Beirut, Riad El-Solh, Beirut P.O. Box 11-0236, Lebanon

[2] Laboratoire Réactions et Génie des Procédés, UMR 7274 CNRS, Université de Lorraine, 54000 Nancy, France.

[3] Laboratory of Applied Studies for Sustainable Development and Renewable Energy (LEADDER), Doctoral School for Science and Technology (EDST), Lebanese University, Campus Rafic Hariri, Hadath, 6573/14, Lebanon.

[4]Laboratory of Materials, Catalysis, Environment and Analytical Methods (MCEMA), Faculty of Science, Lebanese University, Campus Rafic Hariri, Hadath, 6573/14, Lebanon.

Email Address: thibault.roques-carmes@univ-lorraine.fr; joumana.toufaily@ul.edu.lb; mohamad.hmadeh@aub.edu.lb

1. Materials Used for General Synthesis

The linkers including terephthalic acid ($C_6H_4(CO_2H)_2$, 99%) and 2-aminoterephthalic acid ($C_8H_7NO_4$, 99%), as well as the modulator used, namely acetic acid (CH_3COOH, 99%) along with zirconium chloride ($ZrCl_4$, 98%), titanium(IV) isopropoxide ($Ti[OCH(CH_3)_2]_4$, 98%), iron(III) chloride hexahydrate ($FeCl_3 \cdot 6H_2O$, 98%) 1-Naphthylamine, N, N-dimethylformamide (DMF), anhydrous methanol, methanol,ethanol, acetonitrile and sulfuric acid, as well as benzoic acid, 2-propanol, $AgNO_3$, and triethylamine (TEA) used as scavengers, are all acquired from Sigma-Aldrich and Fisher Scientific. With slight modifications, the synthesis of UiO-66 samples is identical to existing protocols.

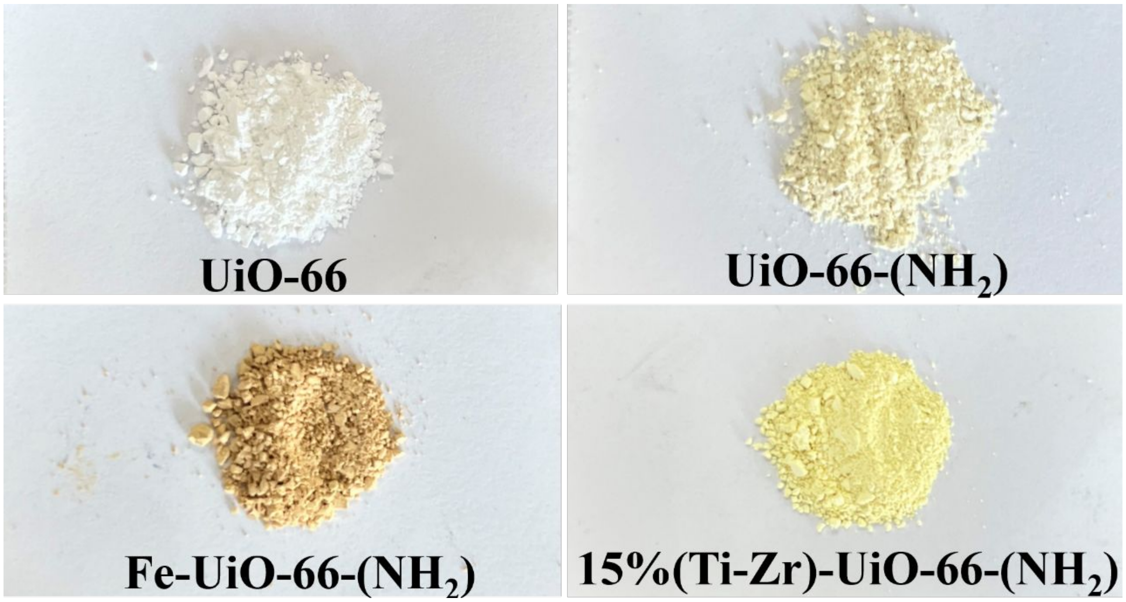

Figure S1. Photographs of MOFs samples.

2. Characterization additional results

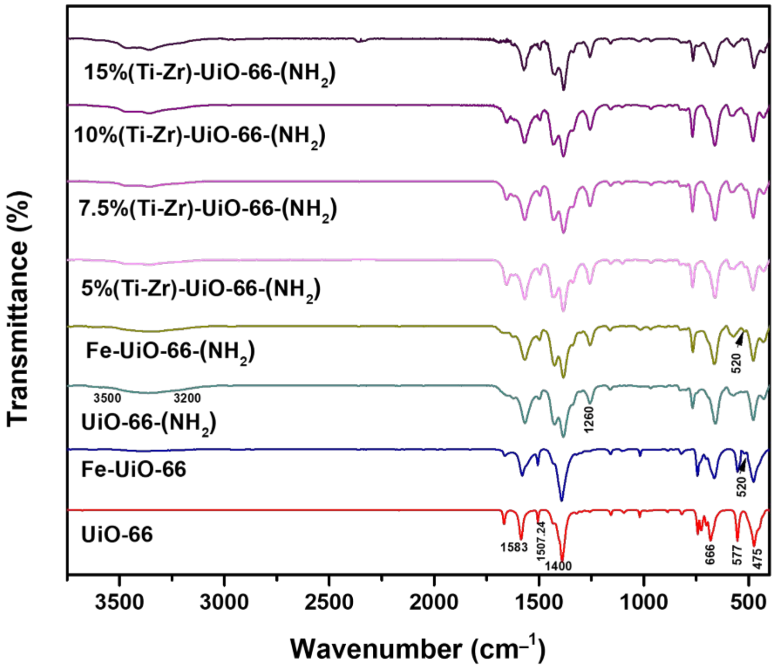

Figure S2. FTIR spectra of the UiO-66-MOFs samples.

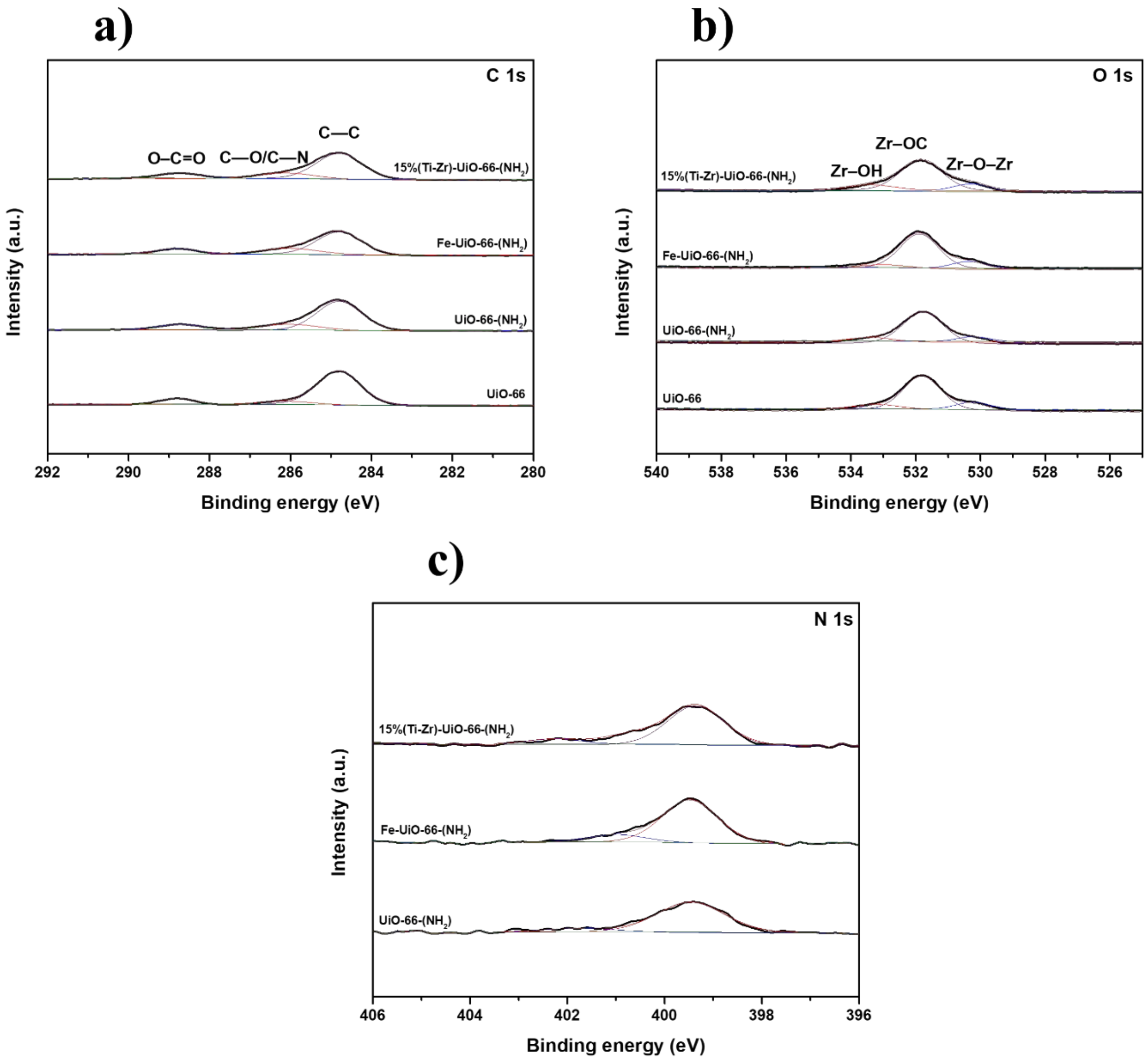

Figure S3. (a) C 1s (b) O 1s and (c) N 1s XPS spectra.

Table S1. Atomic percentages determined by XPS and EDX for 15% Ti-Zr-UiO-66-(NH_2) and Fe-UiO-66-(NH_2).

**Atomic %** / **MOF**	Zr	Ti	Fe	C	O
15%(Ti-Zr)-UiO-66-(NH_2)	3.4<br>8.74	0.71<br>2.94		65.06<br>62.34	26.77<br>25.97
Fe-UiO-66-(NH_2)	4.64<br>2.53		0.5<br>1.08	63.66<br>56.39	27.62<br>40

Red: % from XPS; Blue: % from EDX

Table S2. Surface area, pore volume and number of missing-linker of the samples.

**MOF**	**Surface Area (m^2/g)**	**Pore volume (cm^3/g)**	**Missing-linker defect number from TGA**	**Crystal size (nm)**
UiO-66	1066	0.5	0.87	179
Fe-UiO-66	920	0.4	0.91	174
UiO-66-(NH_2)	972	0.54	1.22	189
Fe-UiO-66-(NH_2)	823	0.5	1.45	194
5%(Ti-Zr)-UiO-66-(NH_2)	976	0.72	1.56	135
7.5%(Ti-Zr)-UiO-66-(NH_2)	958	0.7	1.6	137
10%(Ti-Zr)-UiO-66-(NH_2)	937	0.65	1.85	132
15%(Ti-Zr)-UiO-66-(NH_2)	916	0.55	2.03	138

Table S3. The values of the band gap energy (E_g) in eV of the samples

**MOF**	**Band gap energy (E_g) eV**
UiO-66	3.82
Fe-UiO-66	2.95
UiO-66-(NH_2)	2.86
Fe-UiO-66-(NH_2)	2.36
5%(Ti-Zr)-UiO-66-(NH_2)	2.73
7.5%(Ti-Zr)-UiO-66-(NH_2)	2.65
10%(Ti-Zr)-UiO-66-(NH_2)	2.6
15%(Ti-Zr)-UiO-66-(NH_2)	2.5

3. Photodegradation additional results

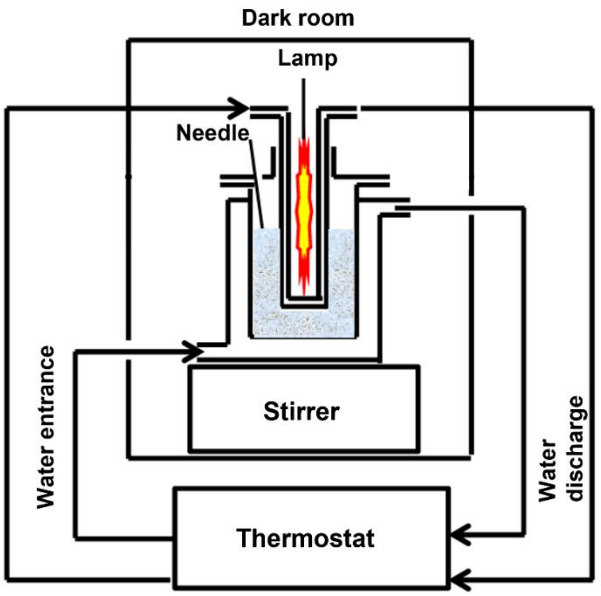

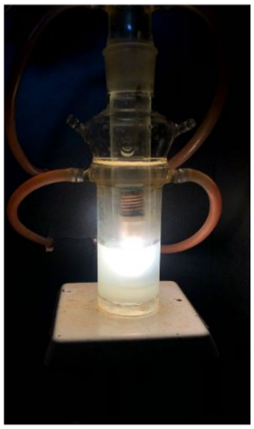

Figure S4. Detailed scheme of the reactor utilized for the 1-NA photodegradation experiments (left) and its real photograph (right).

Pseudo-Order Kinetics

Pseudo-First-Order Kinetics

$$-\ln (C_t/C_0) = k_1 t$$

where:

C_0 is the initial concentration of the pollutant (mg/L),

C_t is the concentration at time t (mg/L),

k_1 is the pseudo-first-order rate constant (min^{-1}),

t is the reaction time (min).

Pseudo-Second-Order Kinetics

$$1/C_t = 1/C_0 + k_2 t$$

where: k_2 is the pseudo-second-order rate constant (L mg^{-1} min^{-1}).

The other parameters are the same as in the first-order equation.

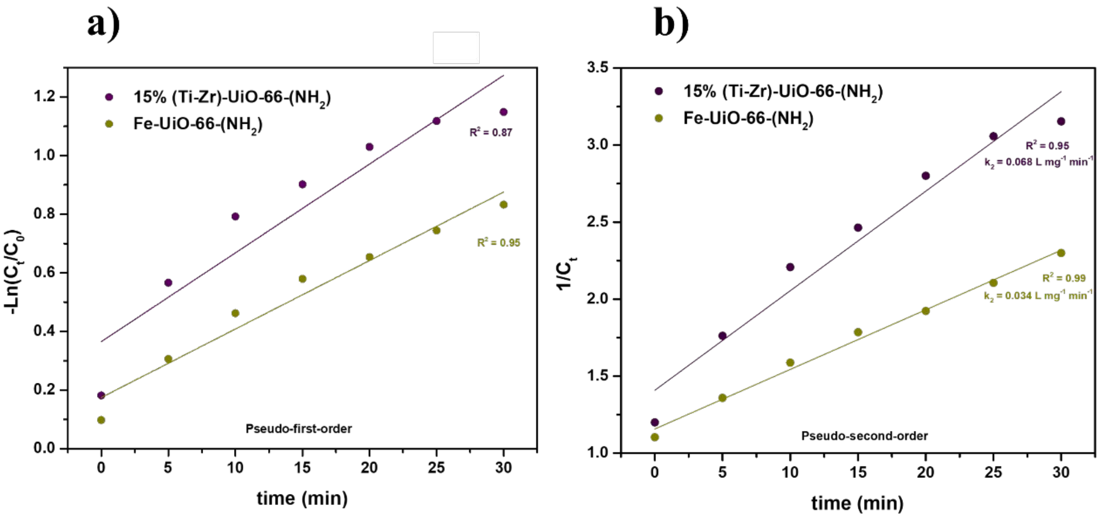

Figure S5. a) Pseudo-first-order and b) pseudo-second order fit for (Ti-Zr)-UiO-66-(NH_2) and Fe-UiO-66-(NH_2) with 1-NA.

Effect of 1-NA concentration and catalyst dosage

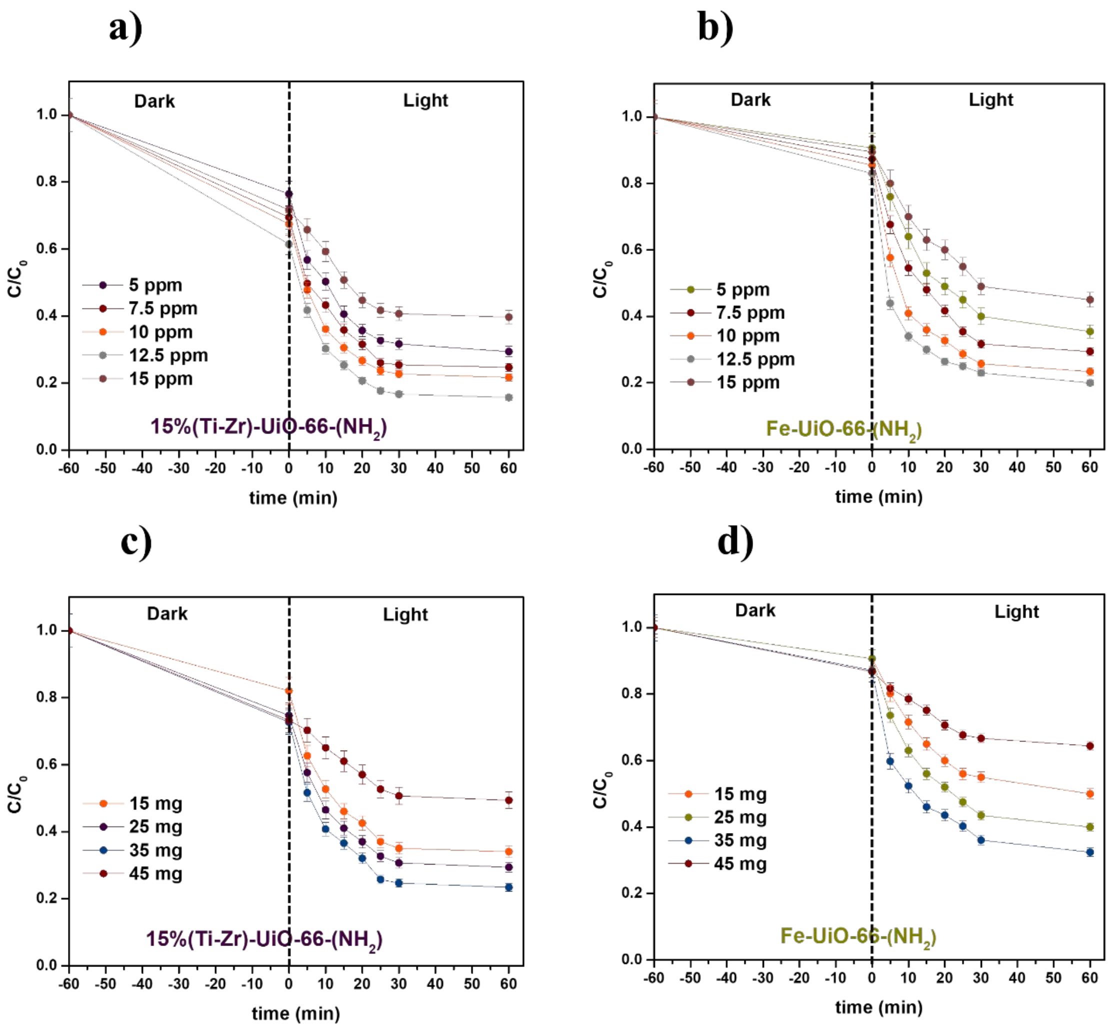

Figure S6. Degradation of 1-NA under a,b) different initial concentration ranging from 5 to 15 ppm and c,d) different catalyst mass ranging from 15 to 45 mg using 15%(Ti-Zr)-UiO-66-(NH_2) and Fe-UiO-66-(NH_2).

1-NA species distribution

For organic acids and bases, the predominance of their protonated or deprotonated forms is determined by the solution's pH relative to their dissociation constant (pKa). When the pH is lower than the pKa, organic acids exist mainly in their non-dissociated form, while organic bases are predominantly in their dissociated form. Conversely, when the pH is higher than the pKa, organic acids favor the dissociated species, whereas organic bases remain mostly in their non-dissociated form.[1]

The fraction of the non-dissociated species for organic acids (f_A^N) and organic bases (f_B^N) can be calculated using the equations:

$$\boldsymbol{f_A^N} = (1+10^{\mathrm{pH - pKa}})^{-1} \quad \text{and} \quad \boldsymbol{f_B^N} = (1+10^{\mathrm{pKa - pH}})^{-1}$$

Similarly, the fraction of the dissociated species for organic acids (f_A^I) and organic bases (f_B^I) is given by:

$$\boldsymbol{f_A^I} = (1+10^{\mathrm{pKa - pH}})^{-1} \quad \text{and} \quad \boldsymbol{f_B^I} = (1+10^{\mathrm{pH - pKa}})^{-1}$$

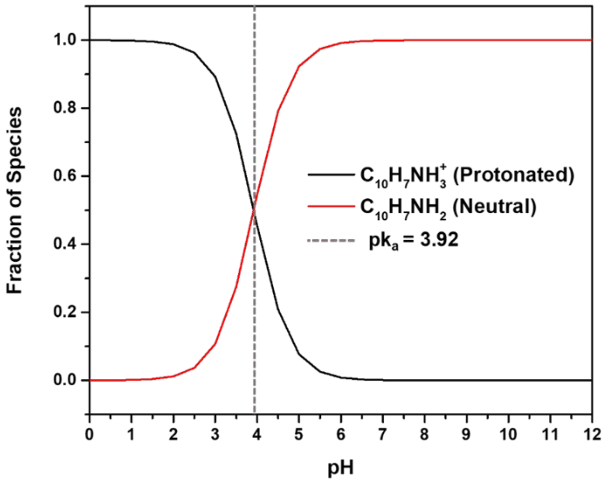

Figure S7. The distribution of 1-NA species.

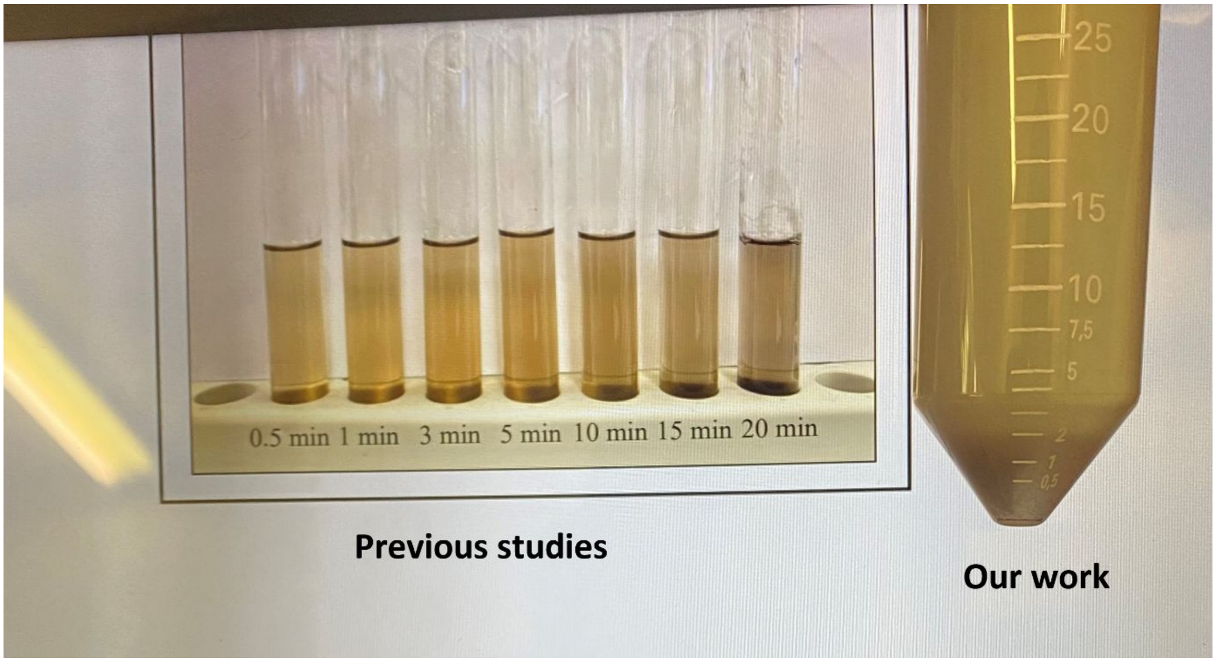

Figure S8. Yellowish solution indicating the presence of pigmented compounds, consistent with findings from previous studies.[2]

UV-vis Spectra

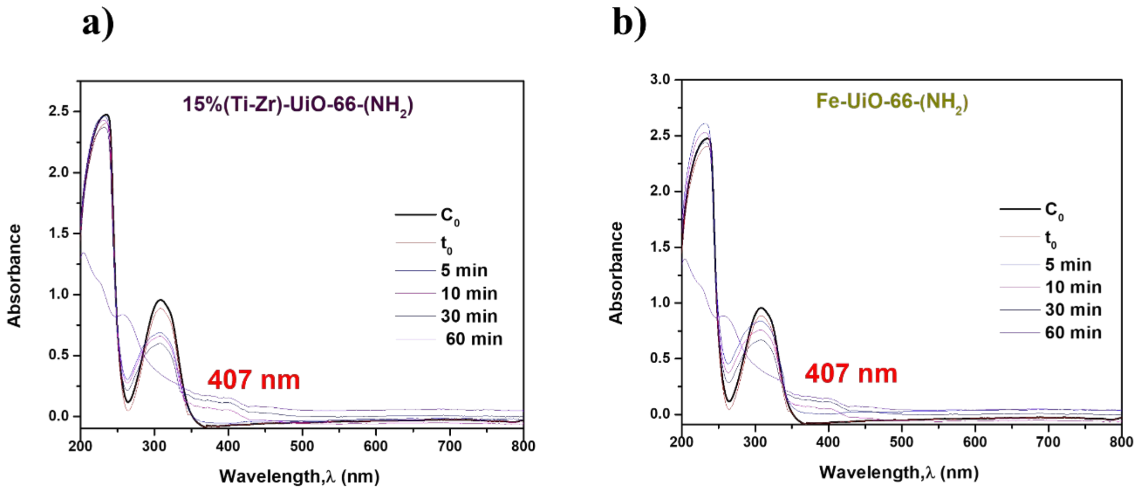

Figure S9. Evolution of UV-vis spectra of 1-NA solutions in the presence of a) 15%(Ti-Zr)-UiO-66-(NH_2) and b) Fe-UiO-66-(NH_2), the peak at 415 correspond to juglone. Experimental conditions: $[1\text{-NA}]_o = 5$ mg L^{-1}; $m_{catalyst} = 25$ mg ; Light Intensity =100W.

HPLC chromatograms

a)

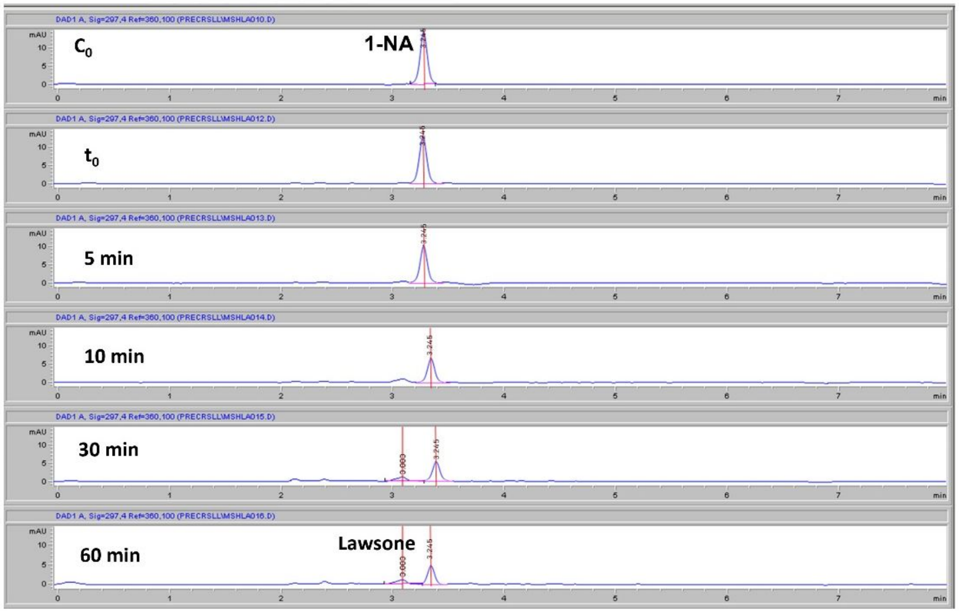

Figure S10. HPLC chromatograms illustrating the 1-NA pattern before and after the photodegradation. Experimental conditions: $[1\text{-NA}]_o$ = 5 mg L^{-1}; $m_{catalyst}$ = 25 mg ; Light Intensity = 100W.

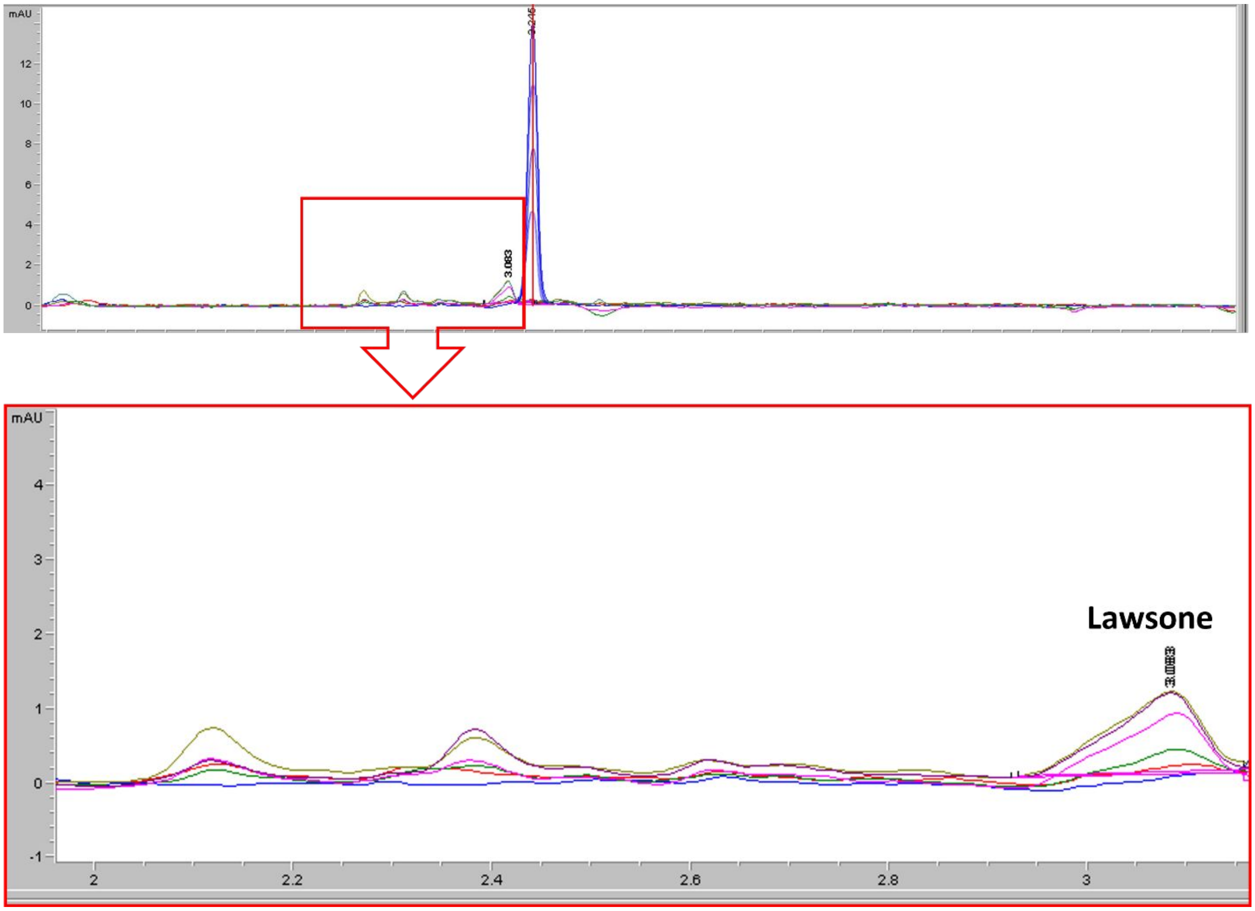

Figure S11. HPLC chromatograms illustrating the lawsone pattern during the photodegradation. Experimental conditions: [1-NA]$_o$ = 5 mg L^{-1}; $m_{catalyst}$ = 25 mg ; Light Intensity = 100W.

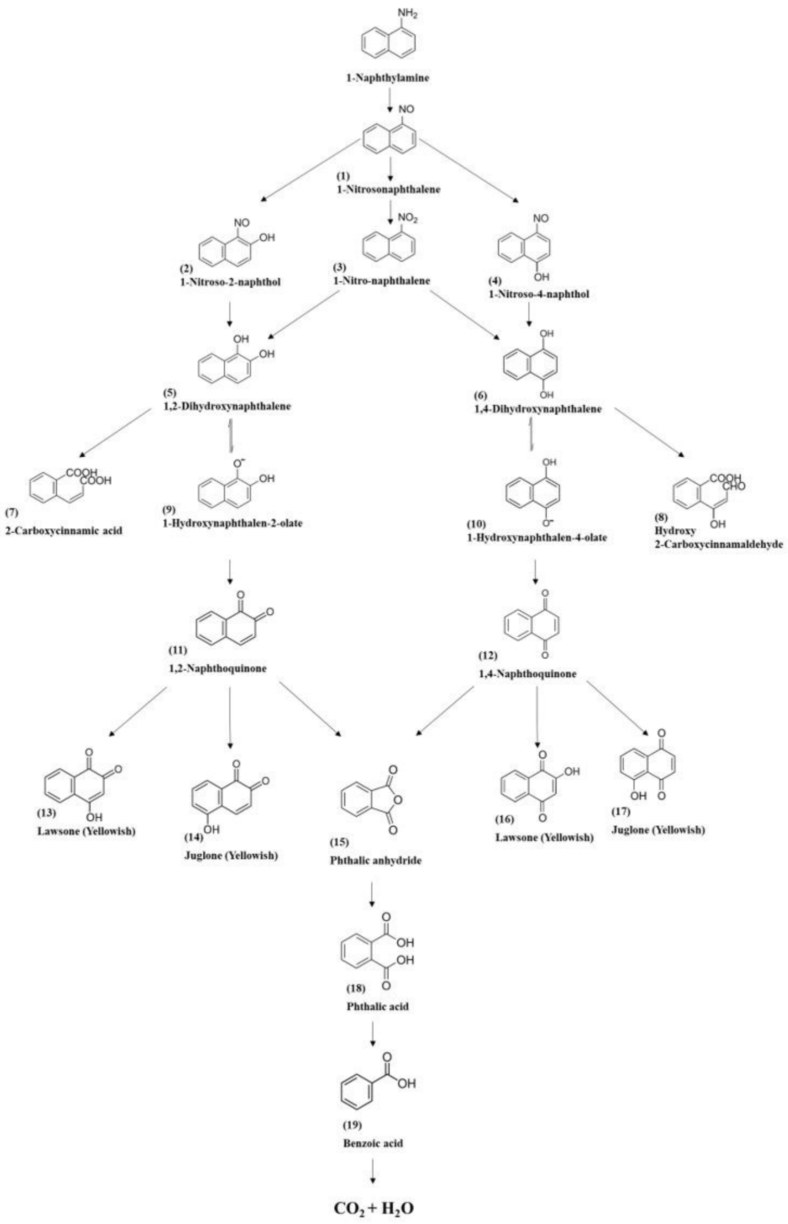

Scheme S1. Proposed mechanism of the degradation of 1-NA.

Table S4: Degradation performance of 1-NA across different systems.

Catalyst	Degradation capacity	Experimental conditions	Ref
UV/Fe^{2+}/PMS	100%	$[1\text{-NA}]_0 = 0.5$ mM, $[Fe^{2+}]_0 = 0.5$ mM, $[PMS]_0 = 1.0$ mM, initial pH = 2.98.	[2]
Nano-MoO_2/PMS	99%	$[1\text{-NA}]_0 = 1$ mg L^{-1}, catalyst dosage= 0.05 g L^{-1}, $[PMS]_0 = 4.0$ mmol L^{-1}, pH = 4.4, 25°C	[3]

4. Post-Photodegradation additional results

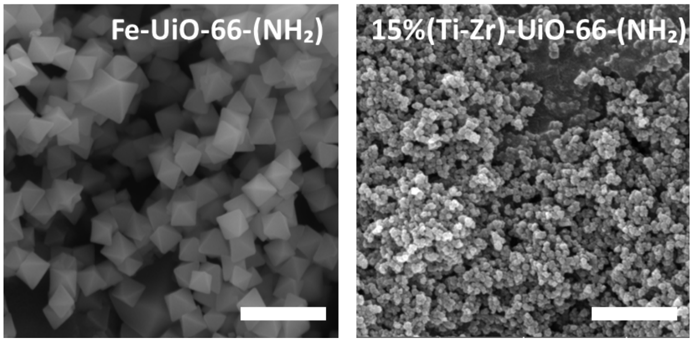

Figure S12: SEM images of Fe-UiO-66-(NH_2) and 15%(T-Zr)-UiO-66-(NH_2) after regeneration (1 µm scale bar).

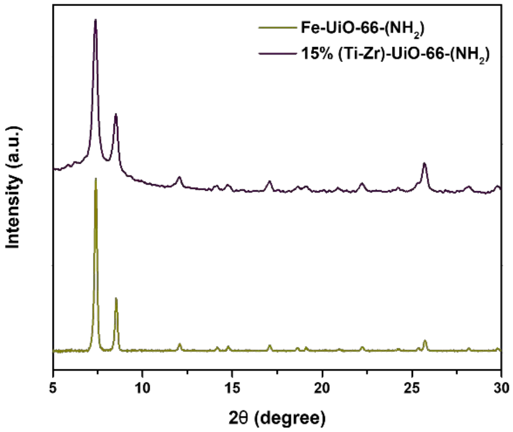

Figure S13: PXRD patterns of Fe-UiO-66-(NH_2) and 15%(T-Zr)-UiO-66-(NH_2) after regeneration.

5. References

1. Yang K, Wu W, Jing Q, Zhu L. Aqueous adsorption of aniline, phenol, and their substitutes by multi-walled carbon nanotubes. Environ Sci Technol. 2008;42:7931–6.

2. Liu J, So HL, Chu W. Degradation of 1-naphthylamine by a UV enhanced Fe^{2+}/peroxymonosulfate system: A novel pH-dependent activation pathway. Chem Eng J. 2022;443:1–30.

3. Chen X, Vione D, Borch T, Wang J, Gao Y. Nano-MoO_2 activates peroxymonosulfate for the degradation of PAH derivatives. Water Res. 2021;192:116834.
